# Dangling bond formation on COF nanosheets for enhancing sensing performances†

**DOI:** 10.1039/d3sc00562c

**Published:** 2023-04-06

**Authors:** Yong-Jun Chen, Ming Liu, Jie Chen, Xin Huang, Qiao-Hong Li, Xiao-Liang Ye, Guan-E. Wang, Gang Xu

**Affiliations:** a State Key Laboratory of Structural Chemistry, Fujian Provincial Key Laboratory of Materials and Techniques Toward Techniques Toward Hydrogen Energy, Fujian Institute of Research on the Structure of Matter, Chinese Academy of Sciences (CAS) Fuzhou Fujian 350002 P. R. China lqh2382@fjirsm.ac.cn gxu@fjirsm.ac.cn; b Institute of Fundamental and Frontier Sciences University of Electronic Science and Technology of China Chengdu 611731 P. R. China; c University of Chinese Academy of Sciences (UCAS) Beijing 100049 P. R. China; d Jiangsu Key Laboratory of Biofunctional Material, School of Chemistry and Materials Science, Nanjing Normal University Nanjing 210023 P. R. China; e Fujian Science & Technology Innovation Laboratory for Optoelectronic Information of China Fuzhou Fujian 350108 P. R. China

## Abstract

Dangling bond formation for COF materials in a rational manner is an enormous challenge, especially through post-treatment which is a facile strategy while has not been reported yet. In this work, a “chemical scissor” strategy is proposed for the first time to rationally design dangling bonds in COF materials. It is found that Zn^2+^ coordination in post-metallization of TDCOF can act as an “inducer” which elongates the target bond and facilitates its fracture in hydrolyzation reactions to create dangling bonds. The number of dangling bonds is well-modulated by controlling the post-metallization time. Zn-TDCOF-12 shows one of the highest sensitivities to NO_2_ in all reported chemiresistive gas sensing materials operating under visible light and room temperature. This work opens an avenue to rationally design a dangling bond in COF materials, which could increase the active sites and improve the mass transport in COFs to remarkably promote their various chemical applications.

## Abstract 2

A facile “chemical scissor” strategy is first proposed to rationally design a dangling bond for COF materials, which remarkably promotes their gas sensing performance.

## Graphical abstract

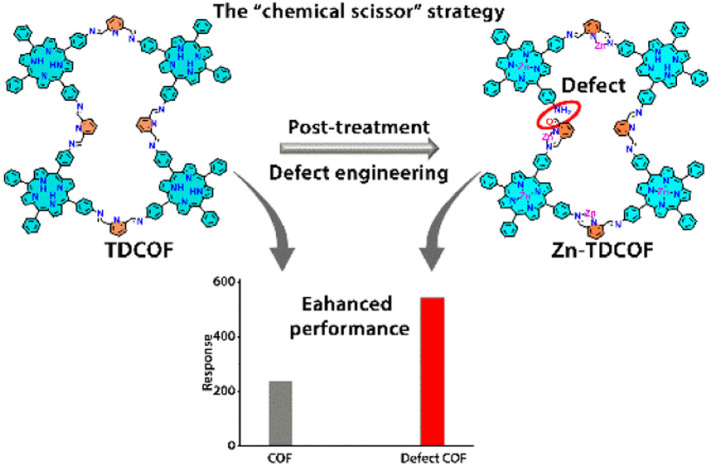

## Introduction

Covalent organic frameworks (COFs), a class of porous crystalline materials constructed from organic molecules by strong covalent bonds, have been widely investigated.^1–3^ Due to their high surface area, diverse structures, superior semiconducting properties and high stability, COFs hold great promise in many applications, like gas storage/separation, catalysis, and sensing.^4–10^ To improve the chemical application of COFs, many strategies such as nanoparticle loading,^11,12^ functional group modification^13,14^ and dangling bond formation^15^ have been developed to increase their active sites. Among them, dangling bond formation is possible to not only increase the active sites, but also create the desired hierarchical pore structures^16^ to improve the mass transfer of COFs in various chemical applications.

Dangling bond formation has played a critical role in the tailoring properties of various materials,^17–20^ which enhances the performances and even generates new properties.^21,22^ In the fields of MOF materials, researchers usually use *de novo* synthesis and post-synthetic treatment to create dangling bonds in MOFs to improve the performance of materials.^23^ However, reports of rational dangling bond formation on COFs are still rare and mainly limited in the use of the monomer-truncation strategy.^15,24–28^ Commonly, this strategy requires introduction of additional monomers in the COF synthesis, which requires twiddly design and preparation.^26^ Comparatively, post-treatment methods, such as ultrasound, soaking (*e.g.*, acid or alkali treatment) and heating, reported for other material systems, are relatively simpler for dangling bond formation.^29,30^ However, the traditional post-treatment methods normally create dangling bonds by randomly breaking the covalent bonds in the framework and cannot achieve rational tailoring of the target bonds. It is still an enormous challenge to realize rational dangling bond formation through a post-treatment method.

One of the biggest advantages of COF materials in performance research is that their porous structure enables effective revelation of the structure–property relationship. In our previous work,^31^ it was revealed that the metal sites at the porphyrin centers of a series of COFs were the critical sensing sites. Co–COF with Co site has the highest NO_2_ adsorption energy and thus shows the highest sensitivity. However, we found a very surprising result in Zn–COF with Zn sites. In the dark, the NO_2_ adsorption energy of Co is much higher than that of Zn, but the sensing response of Co–COF is not as good as that of Zn–COF (for details see the section about the performances of chemiresistive gas sensing). More interestingly, other COF materials in our study showed reversible gas sensing response. Meanwhile, Zn–COF with a relative smaller NO_2_ adsorption energy than other COFs showed a unreversible gas sensing reaction. These unusual results suggest a completely different gas sensing mechanism of Zn–COF from these of other COFs in our previous work.

**Scheme 1:**
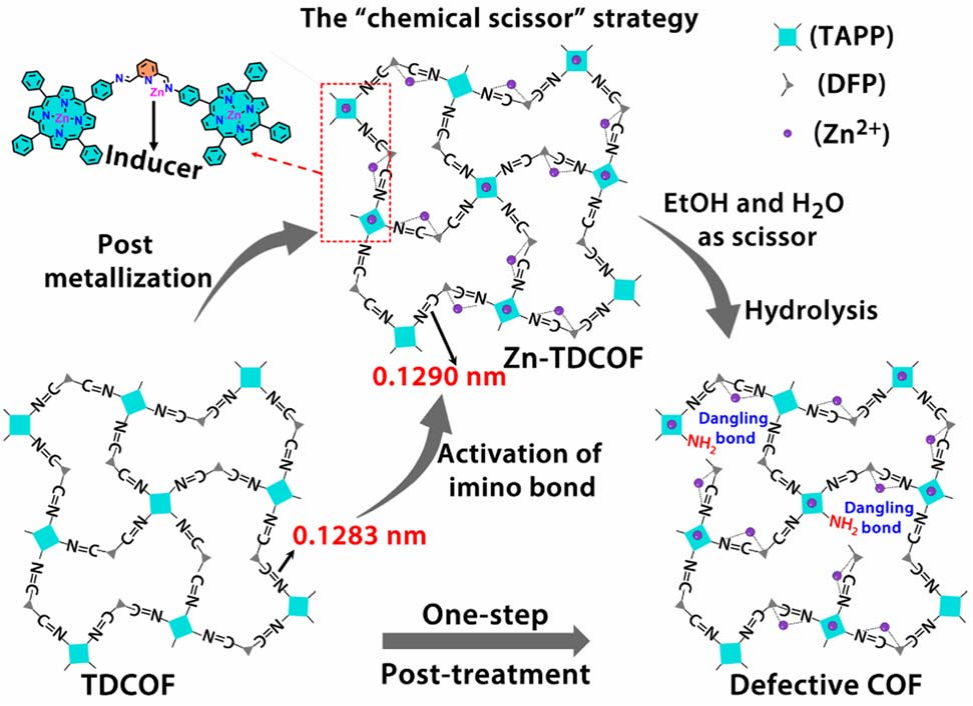
Schematic illustration of the “chemical scissor” strategy for dangling bond formation in TDCOF (two acetate groups coordinated with Zn on pyridine N are omitted for clarity).

In order to further explore the effect of the intrinsic structure–activity relationship of Zn–COF on gas sensing, we propose a facile “chemical scissor” strategy to rationally design a dangling bond for COF materials. The “chemical scissor” strategy works by post-metallizing a COF based on a coordination reaction, which activates the target covalent bond through elongating its lengths to prompt its fracture in the subsequent hydrolysis reaction (Scheme 1). Based on the strategy, metal acts as an “inducer”, and the protonated solvent as a “chemical scissor” to break the target covalent bond. To demonstrate this strategy, TDCOF, constructed with 5,10,15,20-tetrakis(4-aminophenyl)porphyrin (TAPP) and 2,6-diformylpyridine (DFP), was post-treated with Zn^2+^ in a protonic solvent to hydrolyze the target imine bond. The treated sample, Zn-TDCOF-*x* (*x* stands for the treatment time with the unit of an hour) shows the rational fracture of target imine bonds, dramatically increased dangling bonds and hierarchical pore structures. The number of dangling bonds and porosity could be well modulated by controlling the post-treatment time. As an exemplary chemical application, Zn-TDCOF-*x* was used as a chemiresistive gas sensing material to detect NO_2_. Its sensitivity was deeply modulated by controlling the number of dangling bonds and shows one of the highest sensitivities in all reported NO_2_ sensing materials working under visible light and room temperature. A detailed study on the control samples with a thermal gravimetric analyzer (TGA) on a MEMS cantilever, X-ray photoelectron spectroscopy (XPS), Fourier transform infrared (FT-IR) and density functional theory (DFT) calculations reveals the mechanism of dangling bond formation in TDCOF through the “chemical scissor” strategy and the origin of the gas sensing ability from the dangling bonds. Compared with directly obtaining low crystallinity and amorphous materials, the advantage of preparing Zn-TDCOF by the “chemical scissor” strategy are: (1) the porous structure facilitates exploration of the structure–property relationship and gas enrichment; (2) good charge transfer is obtained by reasonable design of dangling bonds.

## Results and discussion

**Fig. 1:**
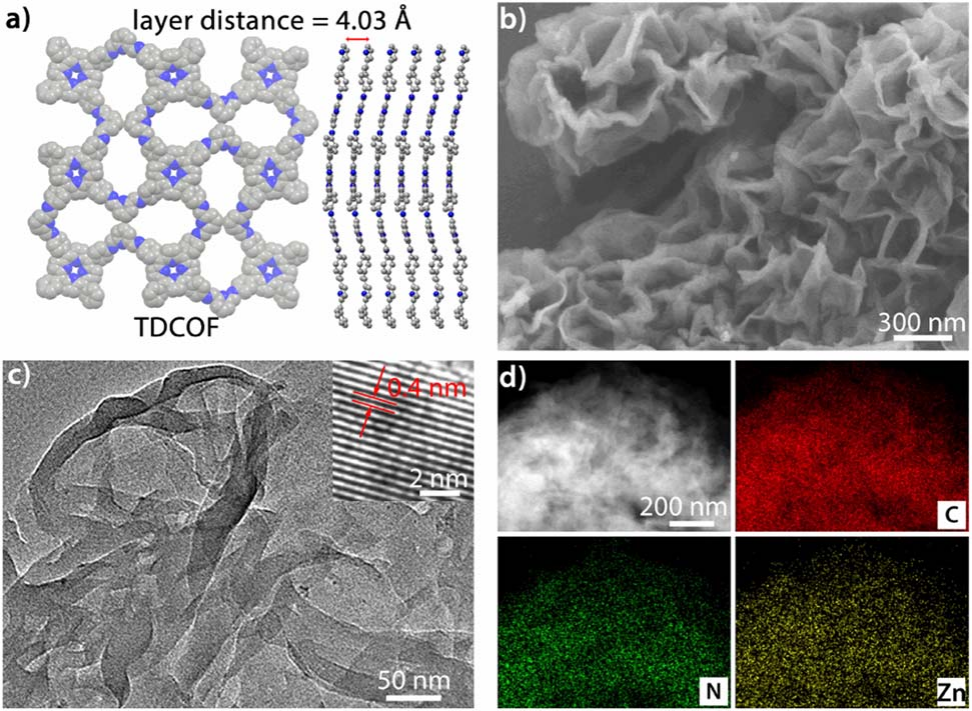
(a) Simulated structure of TDCOF. (b) SEM image of Zn-TDCOF-12. (c) TEM image of Zn-TDCOF-12. (d) Elemental mapping analysis of Zn-TDCOF-12 (C, N and Zn).

TDCOF was synthesized from 5,10,15,20-tetrakis(4-aminophenyl)porphyrin (TAPP) and 2,6-diformylpyridine (DFP) based on a Schiff base reaction under solvothermal conditions (for details see the Method section).^31^ In the crystal structure of TDCOF, one TAPP connects to four DFPs or one DFP connects to two TAPPs through imine bonds to present 2D π-conjugated macromolecular layers. These layers pack with each other at a distance of 0.403 nm (Fig. 1a). The post-modification of TDCOF by Zn^2+^ coordination results in a series of COF materials denoted as Zn-TDCOF-*x*. Powder X-ray diffraction (PXRD) shows that the phases of TDCOF and Zn-TDCOF-*x* are consistent with that of simulated TDCOF (Fig. S1†). The peak (*V*_C=N_ = 1622 cm^−1^) in FT-IR spectra proves the existence of imine linkages of TDCOF and Zn-TDCOF-*x* (Fig. S2†). The high-resolution Zn 2p spectrum (Zn 2p_1/2_, 1044.8 eV and Zn 2p_3/2_, 1021.7 eV)^32^ in XPS demonstrates the successful modification of Zn^2+^ into the structure of Zn-TDCOF-*x*. For example, the Zn 2p_3/2_ binding energy of Zn-TDCOF-12 (1021.7 eV) deviates from that of the reactant, zinc acetate (1022.3 eV), which is attributed to the change in the coordination of Zn species from with oxygen (zinc acetate) to nitrogen atoms (Zn-TDCOF-12) (Fig. S3†).^33,34^ Meanwhile, compared with N 1s of TDCOF, the pyrrolic and pyridinic N in Zn-TDCOF-12 are found to shift obviously, suggesting that they are involved in the coordination with Zn (Fig. S4†).^35,36^ Besides, the content of Zn in Zn-TDCOF-*x* with different treatment times (6, 9, 12 and 15 h) are detected for 0.51, 0.95, 1.37 and 1.82 wt%, respectively, through inductively coupled plasma emission spectrometer (ICP) analyses (Table S1†).

The morphologies of TDCOF and Zn-TDCOF-*x* were characterized through scanning electron microscopy (SEM) and transmission electron microscopy (TEM) measurements. TDCOF presents a 3D crumpled nanosheet morphology. With increase of modification time from 6 to 15 h, the morphologies of Zn-TDCOF-*x* are almost the same as that of TDCOF (Fig. 1b, c and S5–9†) and the thickness of the COF nanosheets is ∼5 nm (Fig. S10†). In the high-resolution TEM image of Zn-TDCOF-12, the lattice fringe spacing (0.4 nm) is close to the layer distance in the simulated TDCOF (Fig. 1a and c). Moreover, the element mapping images reveal that C, N and O are uniformly distributed in Zn-TDCOF-12 and TDCOF (Fig. 1d and S6b†). Compared with TDCOF, Zn-TDCOF-12 shows evenly distributed Zn (Fig. 1d and S6b†). A Zn content of 1.73% (excluding hydrogen) was revealed by energy dispersive X-ray (EDS) analysis, which is basically consistent with the ICP result (Tables S1 and 2†).

The N_2_ sorption tests of TDCOF and Zn-TDCOF were conducted at 77 K to evaluate the porosity. TDCOF exhibits a specific surface area (*S*_BET_) of 813 m^2^ g^−1^ and pore size distribution fitted at 1.1 nm, which matches with the pore size in the simulated structure of TDCOF (1.2 nm) (Fig. S11†). After modifying Zn^2+^, the *S*_BET_ and micropore volume of Zn-TDCOF-*x* are gradually reduced by increasing the treatment time (Fig. S11–14 and Table S3†). Meanwhile, the meso/macro-pore volume is gradually increased. For example, the meso/macro-pore volume of Zn-TDCOF-12 is 4 times higher than that of TDCOF (Table S3†). The above results suggest the successful breaking of the frame structure during the modification process of Zn^2+^ (Table S3†).

**Fig. 2:**
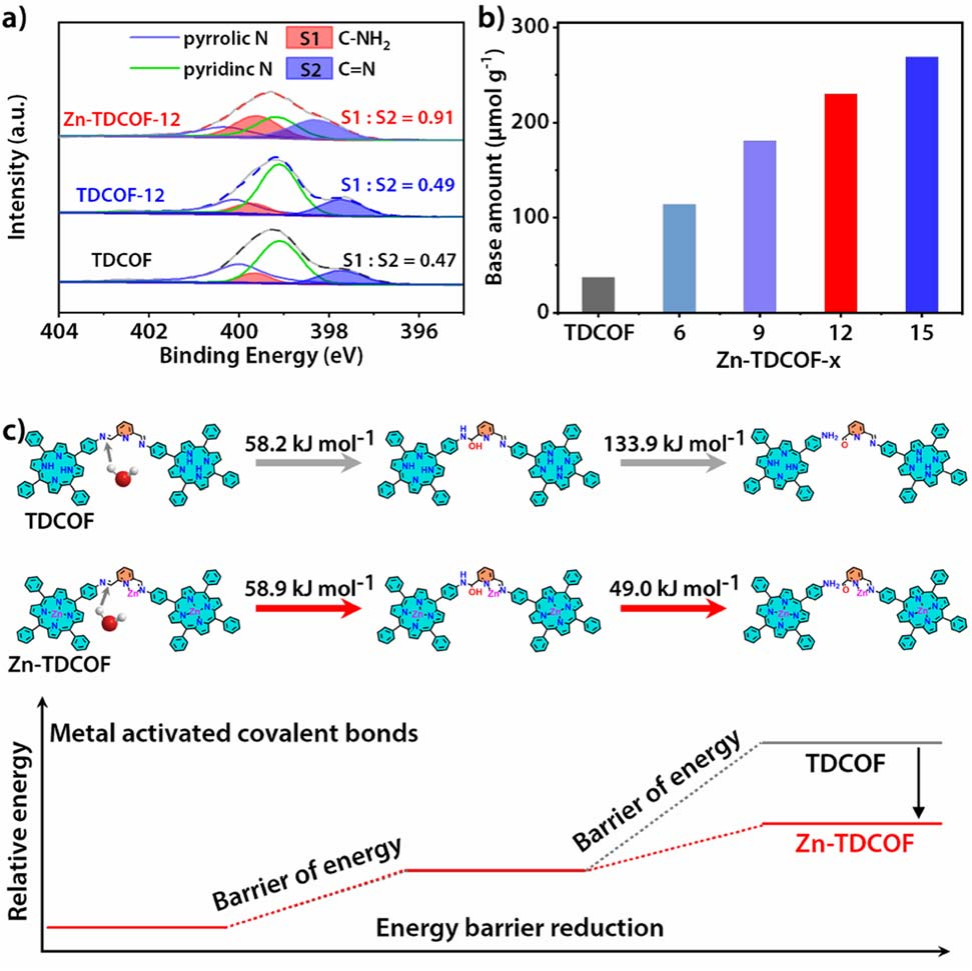
(a) N 1s XPS spectra of Zn-TDCOF-12, TDCOF and pure EtOH treated TDCOF (TDCOF-12) with semi-quantitative analysis for C–NH_2_ and C=N. (b) Number of chemical base sites of TDCOF and Zn-TDCOF-*x* obtained from the chemical adsorption capacity of CO_2_. (c) Energy barriers during dangling bond formation for TDCOF and Zn-TDCOF, respectively.

To further reveal the details of breaking of the frame structure, we analyzed the types of nitrogen in TDCOF and Zn-TDCOF through the semiquantitative analysis of XPS.^37,38^ With the increment of post-treatment time, the ratio of C–NH_2_ and C=N gradually increases (TDCOF: 0.47, Zn-TDCOF-6: 0.57, Zn-TDCOF-9: 0.75, Zn-TDCOF-12: 0.91 and Zn-TDCOF-15: 1.20) (Fig. 2a and S15–17†). The amino groups in TDCOF are from the inherent incomplete structure produced in the COF preparation and the large number of terminal amino groups brought about by the morphology of nanosheets. These results are confirmed by the enhancement of the peak intensity of the amino group (*V*–_NH_2__ = 3300–3500 cm^−1^) in the FT-IR spectrum (Fig. S18†). The amount of –NH_2_ in Zn-TDCOF-*x* was characterized by the test of chemical adsorption of CO_2_ through TGA on a MEMS cantilever (Fig. S19 and 20†). Although the CO_2_ adsorption by the amino group is weak, it is also stronger than physical adsorption and cannot be desorbed within 2 h at room temperature (Fig S19 and 20†). In addition, the CO_2_ adsorption by the small number of unsaturated metal sites in Zn-TDCOF is inconspicuous and desorbed.^39^ Therefore, the non-desorbed CO_2_ is basically adsorbed by the amino group. The method of calculating the number of dangling bonds (–NH_2_) and experimental details can be seen from the quantitative analysis of dangling bonds in the ESI.† With the increase of treatment time, the number of chemical base sites (–NH_2_) increases gradually (Fig. 2b). For example, the number of chemical base sites of Zn-TDCOF-12 (230 μmol g^−1^) is 6.2 times larger than that of TDCOF (37 μmol g^−1^) (Fig. 2b). These results could be explained by the Zn^2+^ coordination prompted damage of the C=N bond and formation of NH_2_ groups in the solvent protonation process.^40^ Thus, post-modification of Zn^2+^ makes TDCOF to expose a large number of dangling bonds. Through Scherrer analysis, we calculated the crystalline domain size of TDCOF and Zn-TDCOF (Table S4†).^41,42^ With the increment of post-treatment time from TDCOF to Zn-TDCOF-15, the domain size gradually decreases (Table S4†). This result coincides with the observation in XPS, FT-IR and chemical adsorption of CO_2_ further confirming the increase of –NH_2_ dangling bonds. In general, the method of introducing amino groups into COF materials is to use small amines as molecular precursors to integrate into the Schiff base framework, but it increases the difficulty of COF preparation.^43^ Dangling bond formation by the “chemical scissor” strategy is relatively simpler, which not only increases the amino groups, but also creates the desired hierarchical pore structures.

The important factors controlling the dangling bond formation of TDCOF were studied as follows. Although it has been reported that the imine bond in other materials could be hydrolyzed by EtOH, PXRD measurements reveal that TDCOF maintains good crystallinity in EtOH for 24 h (Fig. S21a†). However, it completely loses its crystallinity in the EtOH solution of hydrous zinc acetate for the same period. This result suggests that Zn^2+^ is critical in the hydrolyzation reaction and acts as a “inducer” to break the imine bond in the framework of TDCOF to form –NH_2_ groups through the treatment of EtOH and H_2_O as the “chemical scissor” (Fig. 2a, S18 and 22†). The effects of different protonic reagents were studied by using a non-protonic solvent, THF, to replace the protonic solvent, EtOH, to prepare the solution of anhydrous and hydrous zinc acetate, respectively. Different from the phenomenon when using EtOH solution of hydrous zinc acetate as the treatment reagent (Fig. S21b†), the crystallinity of TDCOF is kept partially in THF solution of hydrous zinc acetate for a time longer than 24 h (Fig. S23†), indicating the important role of EtOH in the hydrolyzation reaction. Meanwhile, TDCOF after 12 h treatment in THF solution of anhydrous zinc acetate shows basically unchanged crystallinity and number of dangling bonds (Fig. S24†). This result verifies that, besides EtOH, the lattice water in zinc acetate also contributes to the hydrolyzation reaction.

The dangling bond formation mechanism of Zn-TDCOF was studied through DFT calculations (Fig. 2c and S25†). It is found that after Zn^2+^ metallization, the neighbour imine bond length is increased from 0.1283 to 0.1290 nm (Fig. S25†). The elongated imine bond is activated and easier to broke in the subsequent hydrolysis reaction. Compared with that in TDCOF, the energy barrier of imine bond breaking caused by the solvent attack in Zn-TDCOF-*x* is decreased from 192.1 to 107.9 kJ mol^−1^ (Fig. 2c). These theoretical calculation results implied that Zn^2+^ coordination can effectively prompt the fracture of the neighbour imine bond to produce a dangling bond. Zn^2+^ is only an “inducer” to promote the generation of dangling bonds. We referred to the literature about imine hydrolysis^44^ and analyzed the role of Zn^2+^ in the hydrolysis process through DFT calculations. The possible mechanism of Zn^2+^-promoted imine linkage hydrolysis is shown in Fig. S26.†

Furthermore, we have considered whether other metal ions have a similar function to Zn^2+^ to rationally engineer the dangling bonds. Taking Co–COF for example, the dangling bonds were characterized by a method similar to Zn-TDCOF. After the modification of Co^2+^, a few dangling bonds were also generated, which is proved by the increased ratio of C–NH_2_ and C=N in XPS (Fig. S27†). Compared with original TDCOF (0.47), the ratio in Co–COF is only increased to 0.50, while that in Zn-TDCOF-12 can increase to 0.91 (Fig. 2a and S27†). The different numbers of dangling bonds caused by Co^2+^ (Co-COF) and Zn^2+^ (Zn-TDCOF-12) might be attributed to Zn^2+^ having stronger hydrolysis ability than Co^2+^ to bring more acidic reaction solvent. The increase of dangling bonds caused by post-modification of Co^2+^ is not obvious, which makes it difficult to achieve rational and effective design of dangling bonds for COF materials. In addition, in our previous work, it was revealed that Co^2+^ ions at the porphyrin centers of Co–COF were the critical sensing sites.^31^ Therefore, Co–COF is impossible to explore the structure–activity relationship of dangling bonds and gas sensing.

**Fig. 3:**
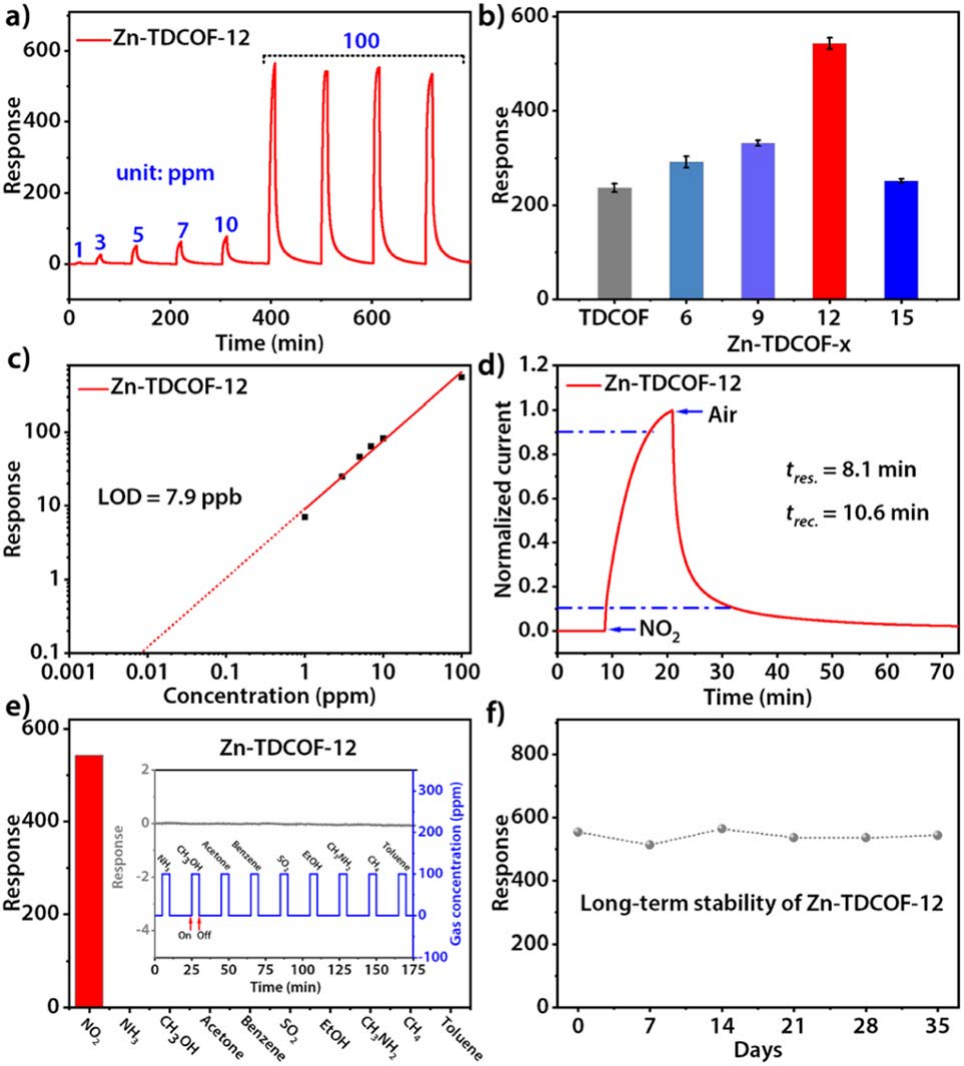
(a) Response–recovery curve toward NO_2_ with different concentrations for Zn-TDCOF-12. (b) Column chart of responses toward 100 ppm NO_2_ of TDCOF and Zn-TDCOF-*x* (*x* = 6, 9, 12 and 15). (c) Response-concentration log–log plots for Zn-TDCOF-12. (d) Response–recovery time curves of 100 ppm NO_2_ for Zn-TDCOF-12. (e) Sensing response of Zn-TDCOF-12 to various gases (100 ppm). (f) Long-term stability of Zn-TDCOF-12 toward 100 ppm NO_2_.

Through post-treatment, the crystalline Zn-TDCOF exposed a large number of dangling bonds as active sites, which would improve the performance in various chemical applications. As an exemplary application, the chemiresistive gas sensing of TDCOF and Zn-TDCOF-*x* was tested using a home-made system.^45^ Zn-TDCOF-12 was taken as an example to explore gas sensing performance in depth. Under dark conditions, the NO_2_ adsorption energy of Co (−1.5 eV) is 6 times higher than that of Zn (−0.25 eV), but the response of Zn-TDCOF-12 to 100 ppm NO_2_ is 4766, which is 1.8 times higher than that of Co–COF in our previous work,^31^ and it can't recover (Fig. S28†). However, it is found that visible light (420–760 nm) irradiation can facilitate the sensing recovery of Zn-TDCOF-12 due to the strong visible-light absorption from the porphyrin functional motif (Fig. 3a and S29†).^33,34^ The average response value of Zn-TDCOF-12 toward 100 ppm NO_2_ is 543 (Fig. 3b), which is one of the highest values for all sensing materials working under visible-light and room temperature reported so far. Under the same test conditions, the response of Co–COF is only 302 (Fig. S30†). Zn-TDCOF-12 also shows a concentration-dependent response to NO_2_ in the range of 1 to 100 ppm (Fig. 3a). The theoretical limit of detection (LOD) can be calculated to be about 7.9 ppb from the simulated linear equation by setting the response to 10% (Fig. 3c). The high signal-to-noise ratio response can be obtained even at a low concentration of NO_2_ (40 ppb), which is lower than the air quality guideline (AQG) level (1 h and 104 ppb) from the World Health Organization (WHO) (Fig. S31 and 32†).

Zn-TDCOF-12 responded to 100 ppm NO_2_ circularly with a low coefficient of variation (2.1%), indicating its good repeatability (Fig. 3a). The response and recovery times are estimated to be 8.1 and 10.6 min, respectively (Fig. 3d). Zn-TDCOF-12 also shows excellent selectivity to NO_2_ against 10 commonly existing interfering gases (Fig. 3e and S33†). After 35 days, about 90% of the original response value to 100 ppm NO_2_ is retained, presenting excellent long-term stability (Fig. 3f). Besides, the structural integrity of Zn-TDCOF-12 remains intact after experiments of long-term stability (Fig. S34†). Notably, it is the first time to show a light-promoted COF chemiresistive gas sensing material. Zn-TDCOF-12 represents one of the best sensing materials for the detection of NO_2_ under visible-light and room temperature conditions (Table S5†).^46–53^

In order to explore the effect of the number of dangling bonds on the gas sensing, the sensing performances of Zn-TDCOF-*x* (*x* = 6, 9, 12 and 15) were compared with each other. With the increment of treatment time, although the *S*_BET_ of Zn-TDCOF-*x* is reduced, their NO_2_ sensitivity displays a Gaussian distribution with a maximum for Zn-TDCOF-12 (Fig. 3b and S35–38†). The sensitivity of Zn-TDCOF-6, Zn-TDCOF-9 and Zn-TDCOF-12 is about 1.3, 1.5 and 2.4 times higher than that of TDCOF (Fig. 3b). Zn-TDCOF-15 with the maximum number of dangling bonds presents a degraded sensitivity, which might be because too many dangling bonds were produced with serious damage of frame structure bonds to restrict the charge transport.^54,55^ Usually, more dangling bonds can bring about more active sites to promote the gas sensing performance. However, the improvement of gas sensing can only be reflected without affecting the charge transfer of materials. When the post-treatment time is increased from 12 to 15 h, the frame structure is severely damaged, which affects the charge transfer to limit the gas sensing. Therefore, Zn-TDCOF-15 shows worse gas sensing than Zn-TDCOF-12. The above results indicate that the “chemical scissor” is a useful strategy to optimize the sensing sensitivity of COF materials by controlling the number of dangling bonds. With the treatment times increasing to 21 h, crystalline Zn-TDCOF cannot be obtained. Zn-TDCOF-21 is an amorphous Zn^2+^-loaded porphyrin-based network (Fig. S21b†), which showed poor response to 100 ppm NO_2_, even lower than that of TDCOF (Fig. S39†). Zn-TDCOF-12 maintained its long-range ordered structure, which exhibited excellent sensing performance (Fig. 3a and S1†). Compared with the disordered structure in the amorphous sample, the long-range ordered structure facilitates electron transfer and provides higher porosity, which are important for improving the sensing performance.

**Fig. 4:**
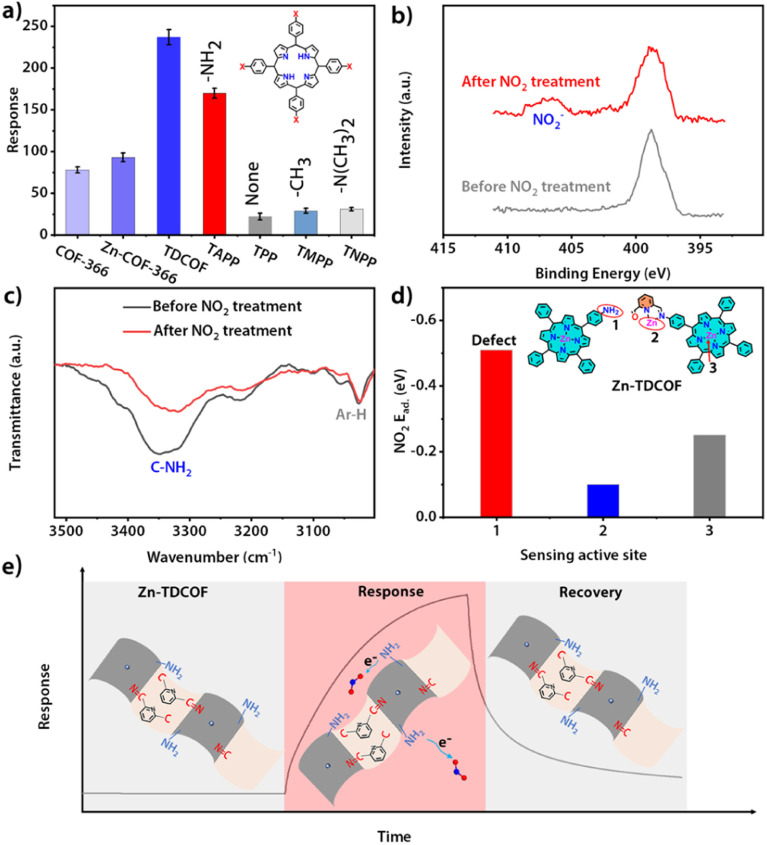
(a) Comparison of the sensing response of various materials to NO_2_. (b) N 1s XPS spectra of Zn-TDCOF-12 before and after NO_2_ (100 ppm NO_2_ and 2 h) treatment. (c) FT-IR spectra of Zn-TDCOF-12 before and after NO_2_ (100 ppm NO_2_ and 2 h) treatment. (d) Comparison of the NO_2_ adsorption energy of the different sites on Zn-TDCOF. (e) Proposed sensing mechanism of NO_2_ sensing for Zn-TDCOF.

The structure of COF-366 is very similar to TDCOF except that 2,6-diformylpyridine (DFP) is replaced by 1,4-phthalaldehyde (PA) (Fig. S40†).^56^ Different from TDCOF, after Zn^2+^ modification, COF-366 does not increase dangling bonds (Fig. S41†). Meanwhile, no obvious improvement in the gas sensing sensitivity of COF-366 is observed (Fig. 4a and S42†). This result indicates that Zn^2+^ is not the main active site for NO_2_ sensing. To further explore the active site of Zn-TDCOF-*x*, the response of porphyrin molecules with different functional groups to 100 ppm NO_2_ was determined. TAPP with the –NH_2_ group presents a higher NO_2_ response than other functional groups (–NH_2_, 170; –H, 47; –CH_3_, 62) (Fig. 4a and S43†). Meanwhile, –N(CH_3_)_2_ in TNPP has the same electron donor capability as –NH_2_ in TAPP to the porphyrin center, but produces a worse response to NO_2_ with a value of 31. These results suggest that –NH_2_ is the main active functional motif for sensing (Fig. 4a). Therefore, the dangling bond formation in Zn-TDCOF not only creates a hierarchical pore structure for better mass transport, but also produces a large number of –NH_2_ groups as the functional motif for NO_2_ sensing, which results in superior gas sensing performances.

Normally, chemisorption by acid–base interaction and physisorption by π-backbonding are the main interactions between the sensing material and NO_2_. Zn–COF-366 shows a recoverable response to 100 ppm NO_2_ under dark conditions, suggesting that it has weak physisorption to NO_2_ and may be due to the interaction between Zn and NO_2_ through π-backbonding (Fig. S44†). However, Zn-TDCOF-12 has an unrecoverable response, suggesting that its adsorption to NO_2_ is strong acid–base chemisorption between –NH_2_ and NO_2_ (Fig. S20, 28 and 45†). The different recovery behaviors of Zn-TDCOF-12 and Zn–COF-366 further confirm that –NH_2_ in Zn-TDCOF-12 is the main functional motif for gas sensing.

XPS and FT-IR measurements further demonstrate that –NH_2_ groups are the functional motif for gas sensing. After Zn-TDCOF-12 was treated with 100 ppm NO_2_ mixed air for 2 h, a new peak belonging to NO_2_^−^ could be observed in FT-IR (1381 cm^−1^) and XPS (407.2 eV) (Fig. 4b and S46†),^57–59^ demonstrating that Zn-TDCOF-12 adsorption of NO_2_ is strong acid–base interaction rather than π-backbonding adsorption. Acid-base interaction is a relatively weak chemisorption, which can induce the reaction to bring about electron transfer.^57–59^ However, the Lewis acid–base interaction usually forms covalent and coordination bonds, which does not undergo electron transfer. The chemical acid–base interaction from ammine groups is different from the Lewis acid–base interaction, which can bring about a redox reaction to form NO_2_^−^. Meanwhile, the peak of –NH_2_ in the FT-IR spectra became weaker after NO_2_ treatment, confirming that –NH_2_ is the main active site (Fig. 4c). Furthermore, we calculated the NO_2_ adsorption energy of porphyrin zinc, pyridine zinc and –NH_2_ (Fig. S47†). Comparatively, –NH_2_ has the strongest adsorption to NO_2_ (Fig. 4d and S47†), confirming that the gas sensitive active site of Zn-TDCOF is –NH_2_, which is exposed to dangling bonds rather than the metal Zn^2+^. In Zn-TDCOF, Zn^2+^ is critical in the hydrolyzation reaction and acts as a “inducer” to promote the fracture of the imine bond in the framework of TDCOF to form –NH_2_ groups. However, Zn^2+^ itself is not a gas-sensitive active site and has no direct correlation with sensing performance. With the comparison experiment of performance, results of XPS and FI-IR spectra of Zn-TDCOF-12 before and after NO_2_ treatment, the dangling bond of –NH_2_ in Zn–COF was revealed as a new type of sensing active site, which is completely different from that in Co–COF.^31^

To explain the sensing function of Zn-TDCOF, we performed a NO_2_ sensing test of other porous materials with amine groups. Taking a representative MOF, NH_2_-UiO-66, for example, the response to NO_2_ was tested under similar conditions to Zn-TDCOF. NH_2_-UiO-66 showed neglectable response to 100 ppm NO_2_ (Fig. S48†), which might be attributed to poor conductivity making charge transfer difficult.^60^ This result illustrates that not all porous materials with amine groups can deliver effective gas sensing performance. We use Zn-TDCOF here is because: (1) highly conjugated porphyrin improves the charge transfer ability of Zn-TDCOF; (2) conjugated 2D layered structure provides electron transport channels; (3) thin nanosheet morphology exposes more gas accessible active sites.

Based on the above discussion, the sensing mechanism can be described as follows (Fig. 4e): (1) Zn-TDCOF-*x* possesses a large number of dangling bonds as gas sensing functional motifs (left part in Fig. 4e); (2) when exposed to NO_2_, –NH_2_ in Zn-TDCOF-*x* transfers electrons to NO_2_ to convert NO_2_ into NO_2_^−^ and produces holes as charge carriers to dramatically increase the current of p-type semiconducting Zn-TDCOF-*x* as a sensing response signal (middle part in Fig. 4e); (3) photo-generated hole oxidates NO_2_^−^ (NO_2_^−^ + h^+^ → NO_2_)^46^ and promotes its desorption from Zn-TDCOF-*x*, which recovers the current to the original (right part in Fig. 4e).

## Conclusions

To rationally design dangling bonds on COF materials, a facile metal coordination induced “chemical scissor” strategy is proposed. This is demonstrated by the post-metallization of Zn^2+^ into the framework of TDCOF nanosheets, which elongates the target imine bond and activated it to bring about the rational fracture of target bonds in a hydrolyzation reaction to create dangling bonds. The number of dangling bonds could be well-modulated by controlling the time for treating TDCOF with Zn^2+^ solution. The critical roles of Zn^2+^, protonic solvent, lattice water of zinc salt, and treatment time played in modulating dangling bonds were also systematically revealed. Compared with pristine TDCOF, Zn-TDCOF-*x* possesses a large number of exposed –NH_2_ dangling groups as chemically active sites and better mass transport avenue. As a result, Zn-TDCOF-12 shows one of the highest sensitivities in all reported NO_2_ sensing materials working under visible light and room temperature conditions. Zn-TDCOF-*x* also shows unique selectivity to NO_2_ among 9 typical interference gases and excellent long-term stability for up to 35 days. This work provides a feasible strategy to rationally modulate the dangling bond for COF materials.

## Methods

### Materials

All solvents and reagents obtained from commercial sources were used without further purification. 5,10,15,20-Tetrakis(4-aminophenyl)porphyrin (TAPP, 98%) was purchased from Shanghai Kylpharma. 2,6-Diformylpyridine (DFP, 98%) and 1,4-phthalaldehyde (PA, 98%) were purchased from Shanghai Aladdin. Zn(OAc)_2_·2H_2_O (AR), acetic acid (AcOH, 99%), ethanol (EtOH, AR) and tetrahydrofuran (THF, AR) were purchased from Sinopharm Chemical Reagent Co., Ltd. 1,2-Dichlorobenzene (AR) was obtained from Shanghai Lingfeng Development Co., Ltd.

### Characterization and instruments

Powder X-ray diffraction (PXRD) patterns of samples were obtained on a Rigaku SmartLab (Japan) with Cu Kα radiation (*λ* = 1.54060 Å). Scanning electron microscopy (SEM, ZEISS Sigma 500) and transmission electron microscopy (TEM, Tecnai F20) were performed to investigate the morphology of the samples. N_2_ adsorption tests were carried out using a BELSORP MAX automatic volumetric gas adsorption analyser and a sample was pretreated at 120 °C for 24 h. The FT-IR spectra were recorded from KBr pellets in the range 4000–400 cm^−1^ on a Nicolet 170 SXFT-IR spectrometer. X-ray photoelectron spectroscopy (XPS) measurements were performed on an American Thermo-VG Scientific ESCALAB 250 Xi XPS system with Al Kα radiation as the exciting source. A xenon arc lamp (PLS-SXE300D) with a light filter (420–760 nm) was utilized as an irradiation source. The test of chemical adsorption of CO_2_ was carried out through a thermal gravimetric analyzer (TGA) on a MEMS cantilever (LoC-TGA-1002, High-End MEMS Technology Co., Ltd).

### Synthesis of TDCOF

The synthesis method of TDCOF follows previously reported procedures.^31^ TAPP (27.1 mg, 0.04 mmol), DFP (10.8 mg, 0.08 mmol), EtOH (0.1 mL), 1,2-dichlorobenzene (0.9 mL) and AcOH (6 M, 0.3 mL) were mixed in a Pyrex tube (o. d × length, 19 × 65 mm and volume, and 10 mL). After sonication for about 25 min, the tube was flash-frozen at 77 K (liquid N_2_ bath) and degassed to achieve an internal pressure of ∼100 mTorr after three freeze–pump–thaw cycles. After the temperature recovered to room temperature, the mixture was heated in an oven at 120 °C for 5 days. After cooling to room temperature, the obtained product was filtered and transferred to a Soxhlet extractor and washed with THF (24 h). After drying at 120 °C under vacuum for 24 h, TDCOF was obtained.

### Synthesis of Zn-TDCOF-*x* (*x* = 6, 9, 12 and 15)

The synthesis of Zn-TDCOF-*x* was carried out with a hydrothermal reaction by Zn metal post-modification based on TDCOF, respectively. Taking Zn-TDCOF-12 for example, Zn(OAc)_2_·4H_2_O (200 mg) was dissolved in 50 mL ethanol solution with sonication for 2 min in a 100 mL flask. The above prepared TDCOF (100 mg) was added to the clear solution and refluxed at 40 °C for 12 h. After cooling to room temperature, the obtained product was washed with water and ethanol each 3 times. After drying at 80 °C under vacuum for 12 h, Zn-TDCOF-12 was obtained.

The synthesis process of Zn-TDCOF-6, 9 and 15 is similar to that of Zn-TDCOF-12 except that the reaction time was different (6, 9 and 15 h), which is denoted as Zn-TDCOF-*x* (*x* = 6, 9, 12 and 15) based on the reaction time. *x* stands for the treatment time for Zn metal post-modification with the unit of an hour.

### Preparation of COF-366 and Zn–COF-366

Firstly, the synthesis method of COF-366 follows previously reported procedures.^56^ TAPP (13.5 mg, 0.02 mmol), PA (7.0 mg, 0.05 mmol), ethanol absolute (1.0 mL), mesitylene (1.0 mL) and acetic acid (6 mol L^−1^, 0.2 mL) were mixed in a Pyrex tube (o. d × length, 19 × 65 mm). The as-obtained COF-366 (100 mg) was added in Zn(OAc)_2_·2H_2_O (200 mg) based ethanol solution (50 mL) followed by sonication for 2 min. The suspension was refluxed at 40 °C for 12 h. After cooling to room temperature, the product was filtered and washed with water and ethanol each 3 times. After drying at 120 °C under vacuum for 12 h, Zn–COF-366 was obtained.

## Data availability

The data are available upon request.

## Author contributions

Y.-J. C. and G. X. conceived the idea. Y.-J. C., M. L., J. C. and X. H. designed the experiments, and collected and analyzed the data. Q.-H. L. accomplished the theoretical calculation. X.-L. Y. and G.-E. W. assisted with the experiments and characterization. Y.-J. C. and G. X. wrote the manuscript. All authors discussed the results and commented on the manuscript.

## Conflicts of interest

There are no conflicts to declare.

## Supplementary Material

SC-014-D3SC00562C-s001
