# Supplementary material for: Dangling bond formation on COF nanosheets for enhancing sensing performances
Source: Chem Sci. 2023 Apr 6;14(18):4824–31. doi: 10.1039/d3sc00562c (PMC10171198; doi:10.1039/d3sc00562c)
Supplement: SC-014-D3SC00562C-s001 [file SC-014-D3SC00562C-s001.pdf]

Electronic Supplementary Material (ESI) for Chemical Science.
This journal is © The Royal Society of Chemistry 2023

Supplementary Information

Dangling Bond Formation on COF Nanosheets for Enhancing Sensing Performances

Yong-Jun Chen,[ac] Ming Liu,[b] Jie Chen,[a] Xin Huang,[d] Qiao-Hong Li,*[a] Xiao-Liang Ye,[a] Guan-E Wang,[a] and Gang Xu*[ace]

[a]*State Key Laboratory of Structural Chemistry, Fujian Provincial key Laboratory of Materials and Techniques toward Techniques toward Hydrogen Energy, Fujian Institute of Research on the Structure of Matter, Chinese Academy of Sciences (CAS), Fuzhou, Fujian, 350002, P. R. China.*

[b]*Institute of Fundamental and Frontier Sciences University of Electronic Science and Technology of China, Chengdu, 611731, P. R. China.*

[c]*University of Chinese Academy of Sciences (UCAS), Beijing 100049, P. R. China.*

[d]*Jiangsu Key Laboratory of Biofunctional Material, School of Chemistry and Materials Science, Nanjing Normal University, Nanjing 210023, P. R. China.*

[e]*Fujian Science & Technology Innovation Laboratory for Optoelectronic Information of China, Fuzhou, Fujian 350108, P. R. China.*

Corresponding author: (Q.-H. Li, E-mail: lqh2382@fjirsm.ac.cn; and G. Xu, E-mail: gxu@fjirsm.ac.cn).

Quantitative analysis of dangling bond in materials.

The amount of dangling bond (-NH_2) was calculated based on the test of the chemical adsorption of CO_2 by the material under room temperature. The CO_2 adsorption was conducted by a thermal gravimetric analyzer (TGA) on a MEMS cantilever. The initial mass (M_0) of the material was obtained based on the D-value of baseline frequency (F_1) and bare cantilever (F_0) on air conditions with a flaw rate of 200 mL min^{-1}. After the material adsorbs high-purity CO_2 for 1 h, the air is introduced to restore it to horizontal frequency (F_2) for 1 h. The mass (M_1) of chemically adsorbed CO_2 was calculated based on the reduction (ΔF) from F_1 to F_2 ($\Delta F = F_1 - F_2$), which cannot be desorbed under room temperature based on the chemical action. The influence of the change in gas density is deducted from all the test data.

The conversion formula from frequency (F) to mass (M):

$$M \text{ (g)} = 10^{-12} \times F \text{ (HZ)}$$

The amount of dangling bond (D) in this work is defined:

$$D = 10^{6} \times M_1/(44 \times M_0) \ \mu\text{mol g}^{-1}$$

Gas sensor characterization

The sensor characterization was conducted by a home-made system reported in our previous work[1]. It takes ~0.65 min to fulfill the quartz chamber when the gas flow was 600 mL min^{-1}. The target gas was introduced into the quartz tube by mixing the certified gas ‘‘mixtures’’ (Beijing Hua Yuan Gas Chemical Industry Co., Ltd., China) and dry air in a proper ratio controlled by the mass flow controllers (CS-200C, Beijing Sevenstar Qualiflow Electronic Equipment Manufacturing Co., Ltd., China) under visible-light irradiation (λ = 420 - 760 nm) and room temperature. The constant flow was 600 mL min^{-1}, the bias on the sensor was 1 V and the current was recorded using Keithley 4200 Sourcemeter.

The sensor response with a positive response in this work is defined as the ratio of sensor resistance in the air (R_{air}) and analytic gas (R_{analyte}):

$$\text{Response} = R_{air}/R_{analyte} - 1$$

The response time ($t_{res.}$) of the sensor with a positive response is the time required for the increasing current to 90% of the saturation value and the recovery time ($t_{rec.}$) is the time required to decrease the saturated current to its 10%.

The coefficient of variation (CV) is used to represent the change of different cycles

on responses, which is defined as:

$$CV = R_{SD}/R_{average} \times 100\%$$

R_{SD} and $R_{average}$ are the standard deviation (SD) and an average value of responses with different cycles (100 ppm NO_2), respectively.

Computational details.

All calculations were performed using the DFT approach implemented in Dmol3 package.[2-3] The generalized gradient approximation (GGA) was adopted to describe the density function using the Perdew-Burke-Ernzerhof (PBE) functional for the exchange-correlation term. The double-numeric quality basis set with polarization functions (DNP)[2, 4-5] was adopted, which was comparable to 6-31G**.[6-7] The numerical basis sets can minimize the basis-set superposition error.[8] A Fermi smearing of 0.005 hartree was utilized for structural relaxation and TSs location. The tolerances of the energy, gradient and displacement convergence were 1×10^{-5} hartree, 2×10^{-3} hartree/Å, and 5×10^{-3} Å, respectively. The transition states (TS) were located using the complete linear synchronous transit/quadratic synchronous transit (LST/QST) methods. The structure of TDCOF was extracted from the crystal structure.

The adsorption energy with zero-point-corrected $\Delta E_{ads.}$ is defined as

$$\Delta E_{ads.} = [E_{total} - (E_{slab.} + E_{adsorbate})] + \Delta ZPE_{ads} \quad \text{(E1)}$$

$$\Delta ZPE_{ads.} = \left(\sum_{i=1}^{Vibrations} \frac{hv_i}{2}\right)_{adsorbed} - \left(\sum_{i=1}^{Vibrations} \frac{hv_i}{2}\right)_{gas} \quad \text{(E2)}$$

Where E_{total}, E_{slab} and $E_{adsorbate}$ represented the total energy of the slabs with adsorbate, the bare slab of the surface, and the free adsorbate molecules, respectively. ΔZPE_{ads} referred to the zero-point vibrational energy (*ZPE*) correction for the adsorption. E2 was used to calculate the ZPE correction via the vibrational frequencies for the species, including the gas phase and the adsorbed state, where *h* was Planck's constant and v_i represented the vibrational frequency. With the definition of adsorption energy, more negative values reflected the strong interactions between the adsorbed species and the slab surface.

For a reaction such as R(reactant) → P(product), the activation barrier with the zero point-corrected (ΔE_a) was calculated according to equations (E3) and (E4):

$$\Delta E_a = (E_{TS} - E_R) + \Delta ZPE_{barrier} \quad \text{(E3)}$$

$$\Delta ZPE_{barrier} = \left(\sum_{i=1}^{Vibrations} \frac{hv_i}{2}\right)_{TS} - \left(\sum_{i=1}^{Vibrations} \frac{hv_i}{2}\right)_{R} \quad \text{(E4)}$$

Where E_R and E_{TS} were the total energies of the reaction and transition state, respectively. $\Delta ZPE_{barrier}$ referred to the ZPE correction for the reaction barrier. In E4, the front term included the vibrational frequencies of the species in the TS, in which the imaginary frequency was not considered, and the rear term included the vibrational frequencies of the adsorbed reactions.

The reaction energy with the zero point-corrected (ΔE) was calculated according

to E5 and E6:

$$\Delta E = (E_P - E_R) + \Delta ZPE_{energy} \quad \text{(E5)}$$

$$\Delta ZPE_{energy} = \left(\sum_{i=1}^{Vibrations} \frac{hv_i}{2}\right)_P - \left(\sum_{i=1}^{Vibrations} \frac{hv_i}{2}\right)_R \quad \text{(E6)}$$

Where E_R and E_P were the total energies of the reaction and product, respectively. ΔZPE_{energy} referred to the ZPE correction for the reaction energy, determined by the vibrational frequencies of the reactions and products.

In order to eliminate the interactions from molecules in the neighboring periodic box, the length, width and height of the periodic rectangle boxes were set to be 25, 25 and 14 Å, respectively. All the atoms were unfixed during the structural optimization. Before structural optimization, all the initial structures of adsorbates-substrate were made by connecting the most reactive parts of them. The associated reactivities were calculated by the spatial Fermi softness.[9] The adsorption energy $E_{ad.}$ was calculated via:

$$E_{ad.} = E_{A*} - E_A(g) - E^*$$

where E_{A*}, $E_A(g)$ and E^* represent the DFT energies of adsorbates with substrates, gaseous adsorbates and the substrates, respectively.

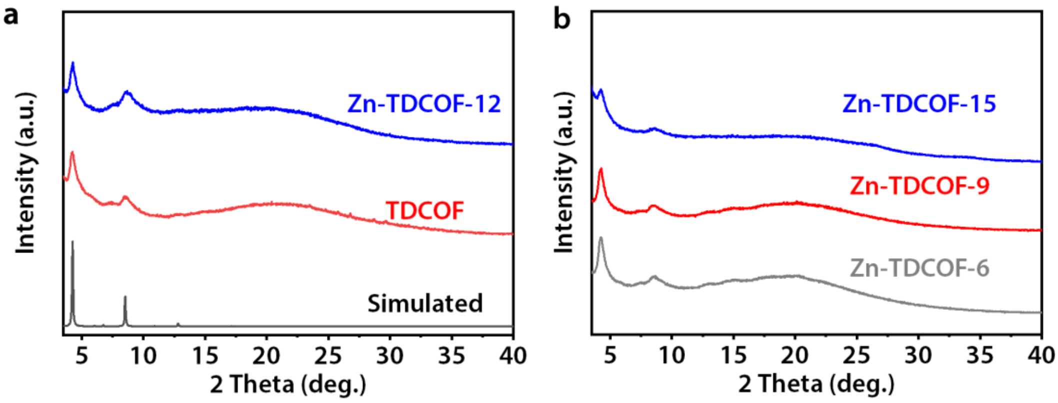

Fig. S1 PXRD patterns of TDCOF and Zn-TDCOF-x (x = 6, 9, 12 and 15).

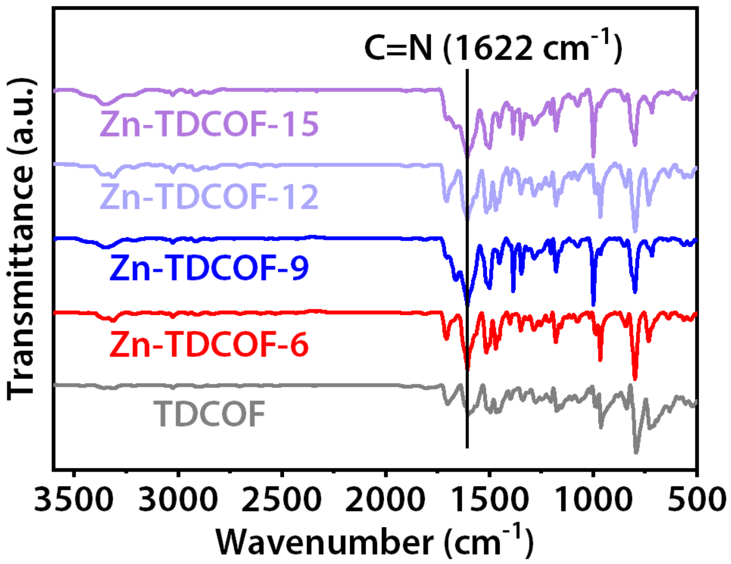

Fig. S2 FT-IR spectra of TDCOF and Zn-TDCOF-x (x = 6, 9, 12 and 15).

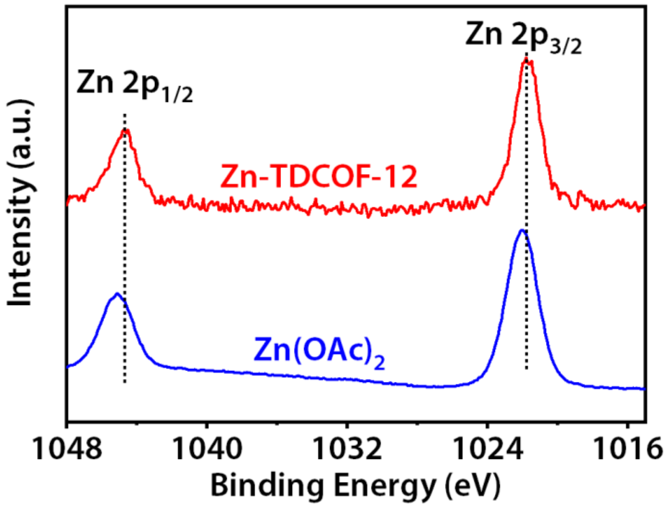

Fig. S3 Zn 2p XPS spectra of $Zn(OAc)_2$ and Zn-TDCOF-12.

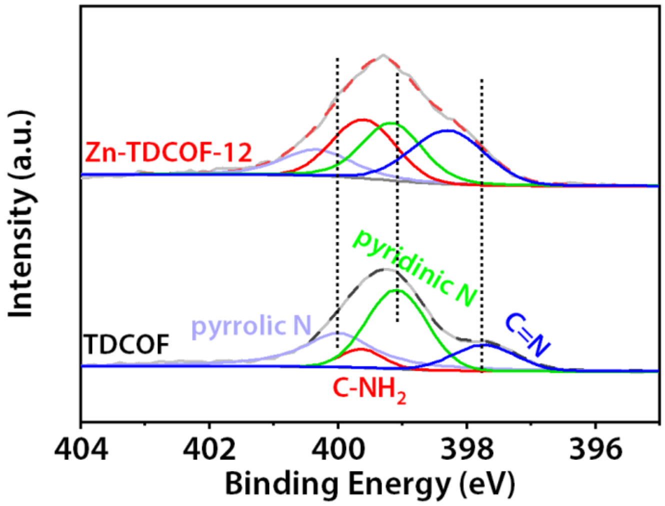

Fig. S4 N 1s XPS spectra of TDCOF and Zn-TDCOF-12.

Table S1. Zn element content of Zn-TDCOF-x (x = 6, 9, 12 and 15).

Sample	Found (wt%)
Zn-TDCOF-6	0.51%
Zn-TDCOF-9	0.95%
Zn-TDCOF-12	1.37%
Zn-TDCOF-15	1.82%

Table S2 Element content (excluding hydrogen) of Zn-TDCOF-12 by Energy dispersive X-ray (EDS) analysis.

Element	C	N	Zn
Content (wt%)	85.56	12.72	1.73

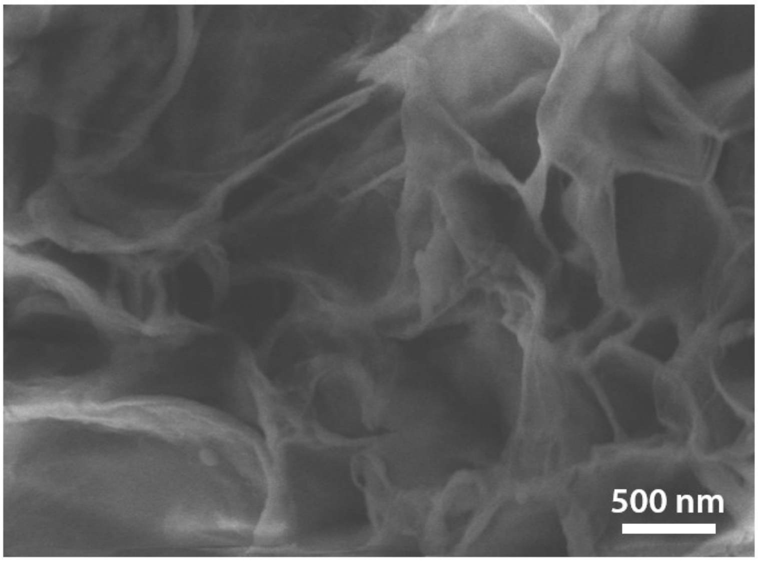

Fig. S5 SEM image of TDCOF.

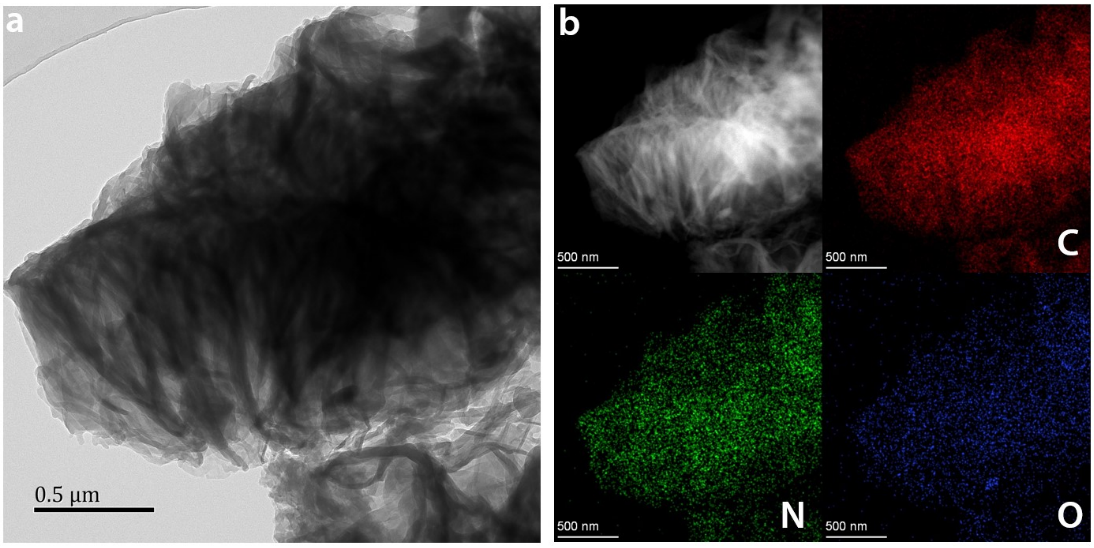

Fig. S6 Morphology of TDCOF. **a** TEM image. **b** Elemental mapping analysis (C, N and O).

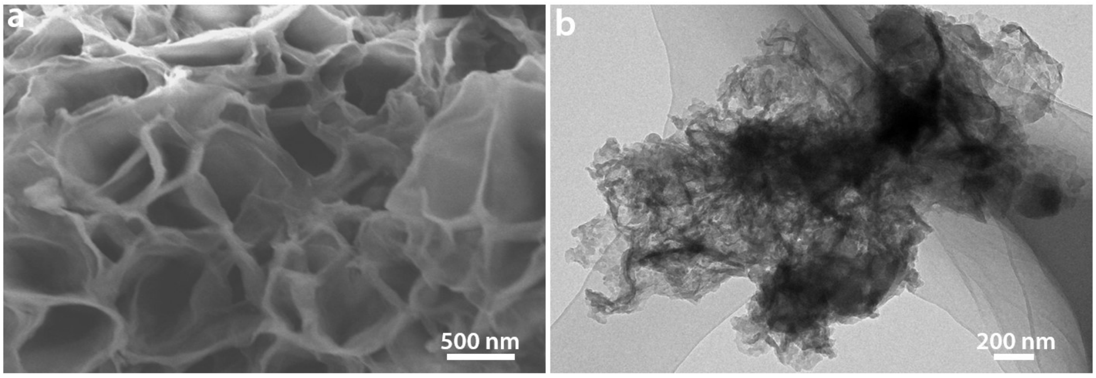

Fig. S7 Morphology of Zn-TDCOF-6. **a** SEM image. **b** TEM image.

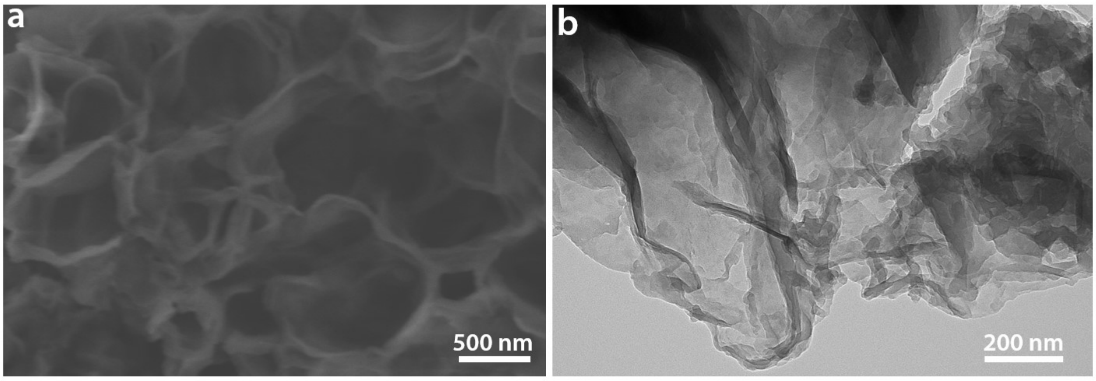

Fig. S8 Morphology of Zn-TDCOF-9. **a** SEM image. **b** TEM image.

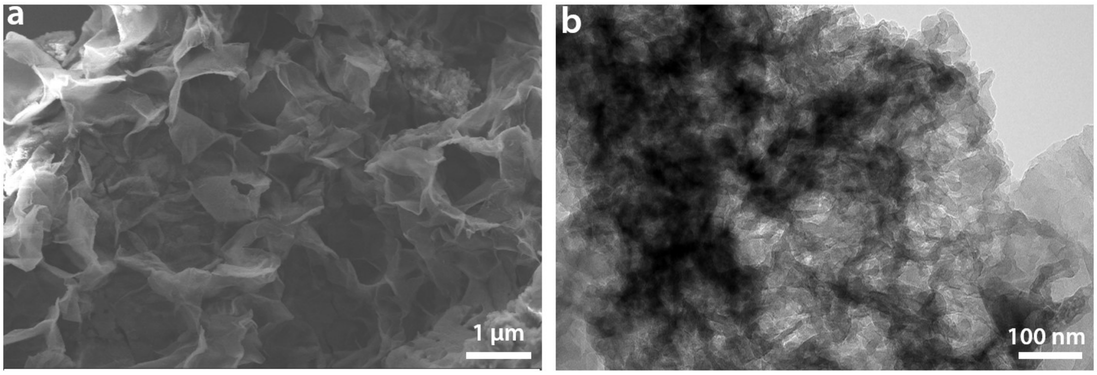

Fig. S9 Morphology of Zn-TDCOF-15. **a** SEM image. **b** TEM image.

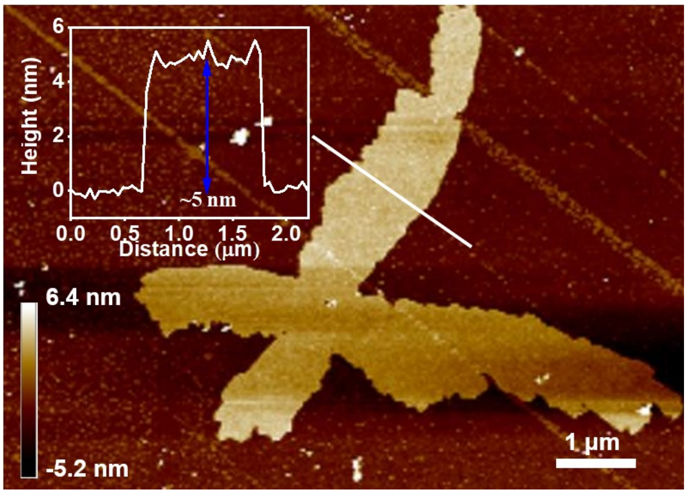

Fig. S10 AFM image of Zn-TDCOF-12.

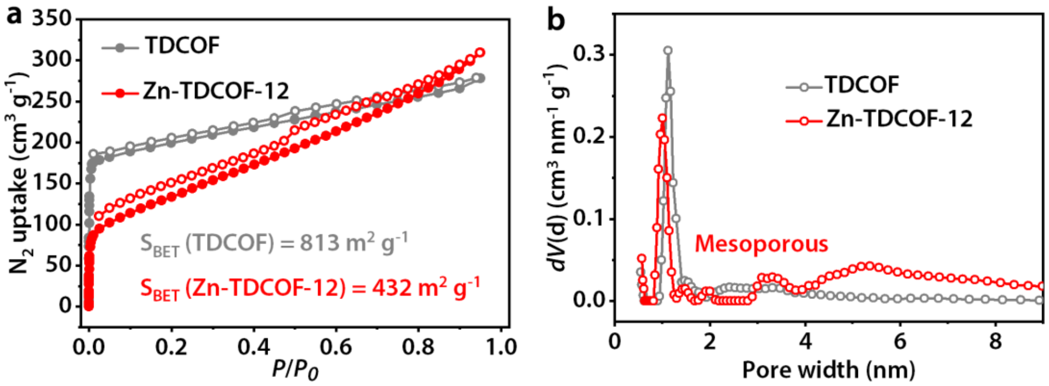

Fig. S11 N_2 sorption tests of TDCOF and Zn-TDCOF. **a** N_2 sorption curves. **b** Pore size distribution curves.

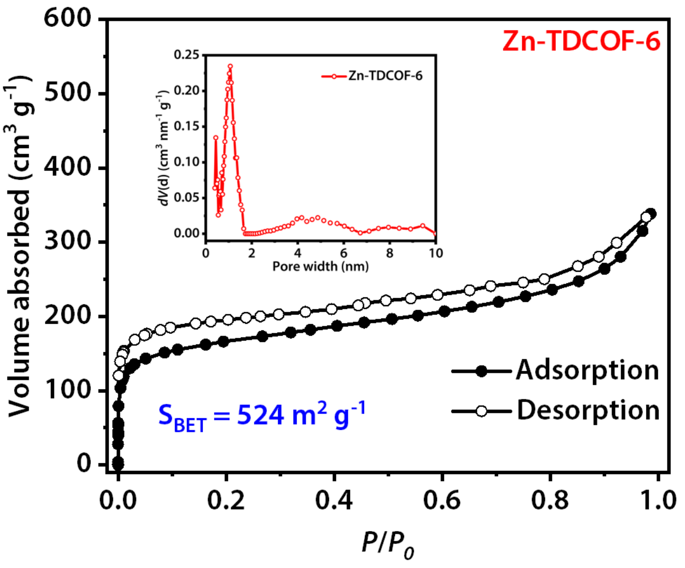

Fig. S12 N_2 sorption and pore size distribution curve of Zn-TDCOF-6.

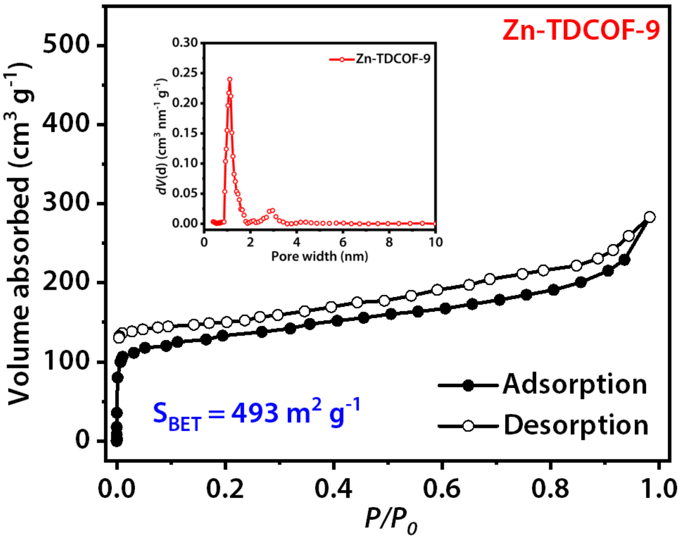

Fig. S13 N_2 sorption and pore size distribution curve of Zn-TDCOF-9.

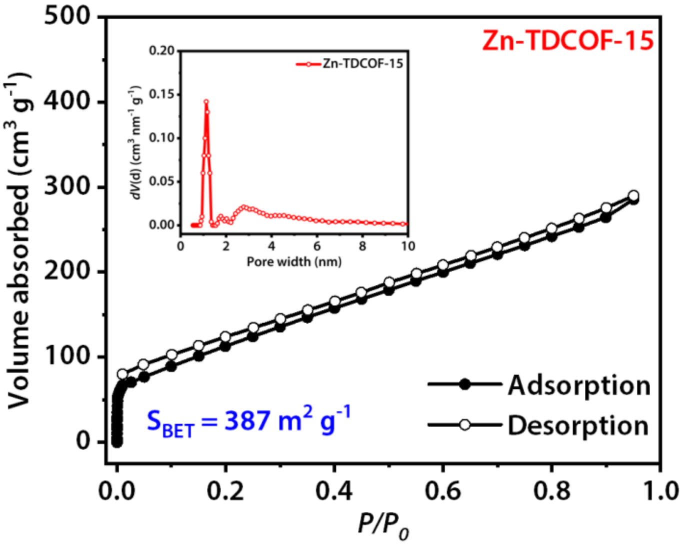

Fig. S14 N_2 sorption and pore size distribution curve of Zn-TDCOF-15.

Table S3 S_{BET} and pore distribution of TDCOF and Zn-TDCOF-x (x = 6, 9, 12 and 15).

	S_{BET} (m^2 g^{-1})	V_{micro} (cm^3 g^{-1})	$V_{meso/macro}$ (cm^3 g^{-1})
TDCOF	813	0.326	0.049
Zn-TDCOF-6	524	0.224	0.112
Zn-TDCOF-9	493	0.194	0.134
Zn-TDCOF-12	432	0.115	0.197
Zn-TDCOF-15	387	0.082	0.201

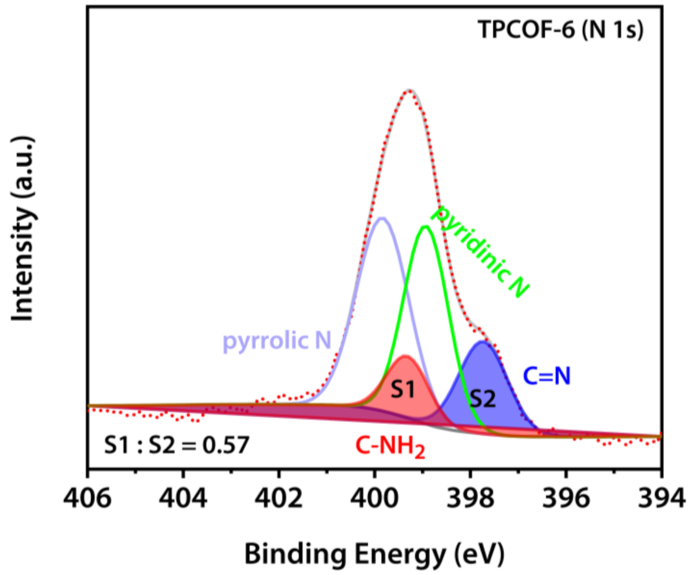

Fig. S15 N 1s XPS spectrum of Zn-TDCOF-6 with semi-quantitative analysis for C-NH_2 and C=N.

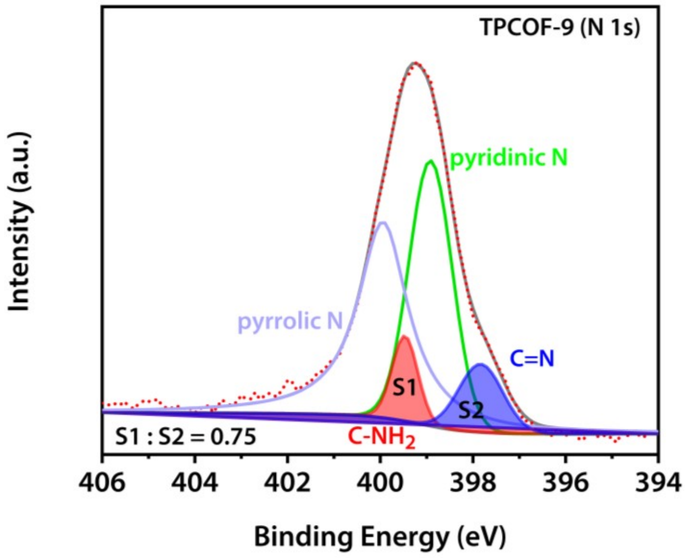

Fig. S16 N 1s XPS spectrum of Zn-TDCOF-9 with semi-quantitative analysis for C-NH_2 and C=N.

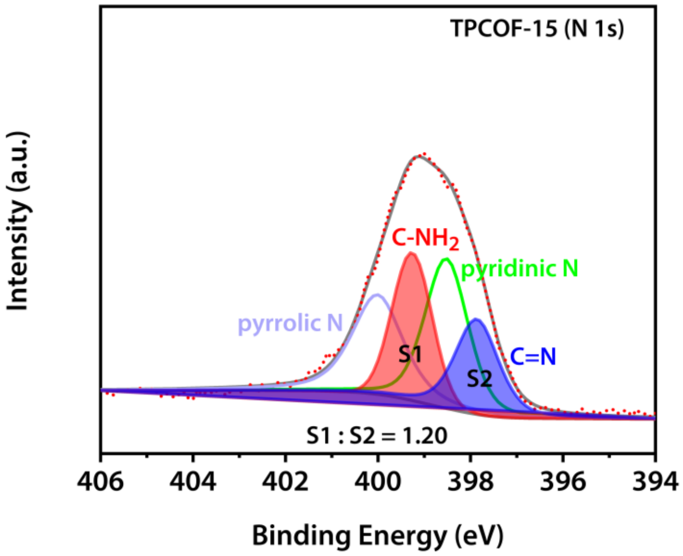

Fig. S17 N 1s XPS spectrum of Zn-TDCOF-15 with semi-quantitative analysis for C-NH_2 and C=N.

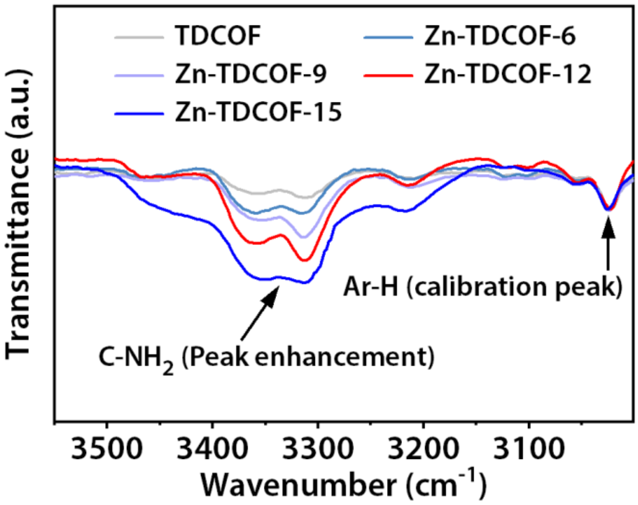

Fig. S18 FT-IR spectra of TDCOF and Zn-TDCOF-x (KBr pellet: 2 mg samples dispersed in 200 mg KBr).

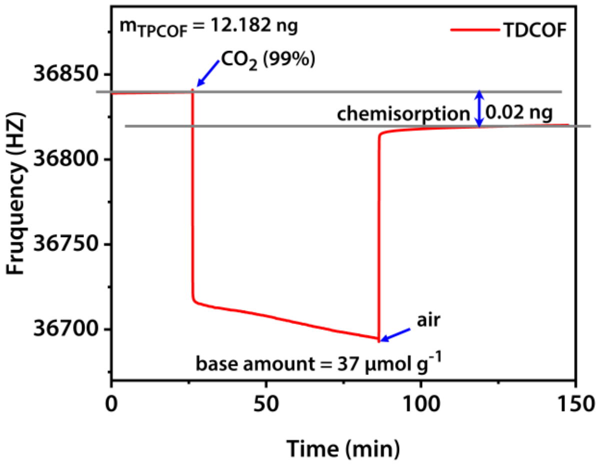

Fig. S19 CO_2 adsorption of TDCOF based on the TGA on a MEMs cantilever to calculate the amount of chemical base sites.

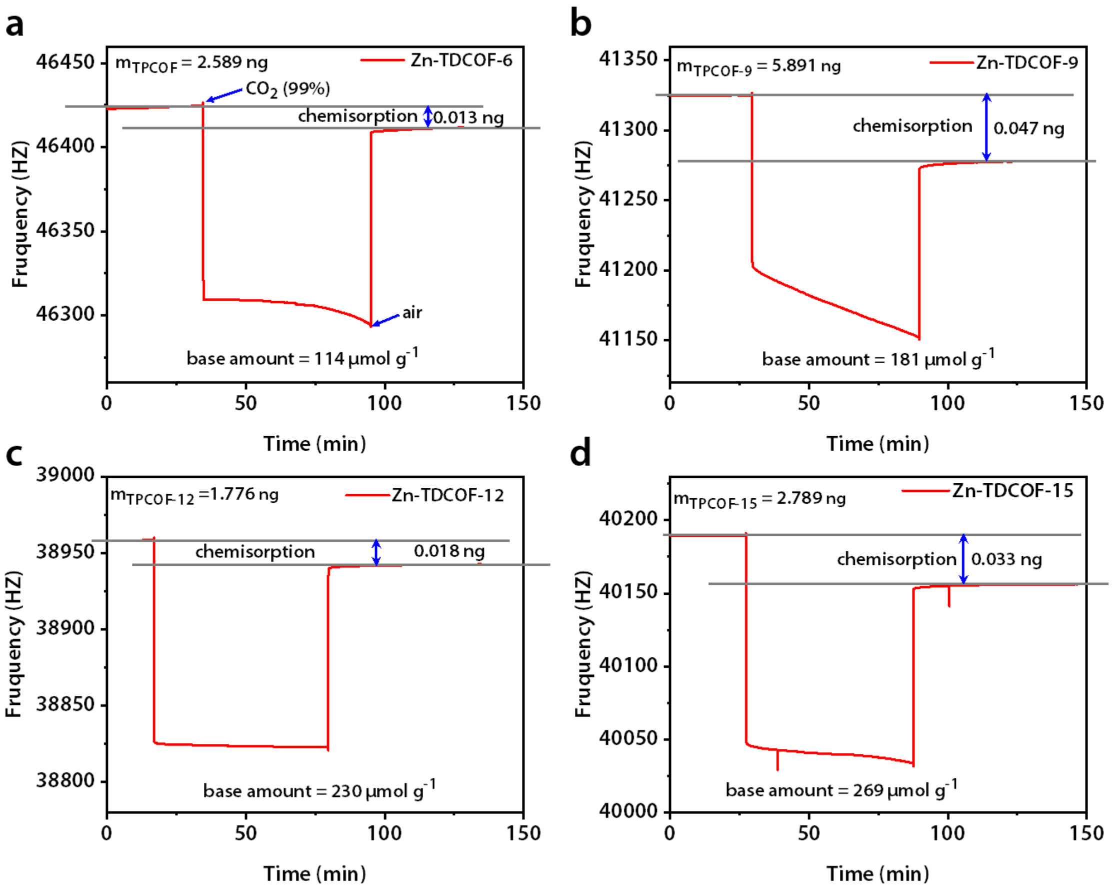

Fig. S20 CO_2 adsorption of Zn-TDCOF-x based on the TGA on a MEMs cantilever to calculate the amount of chemical base sites. **a** Zn-TDCOF-6. **b** Zn-TDCOF-9. **c** Zn-TDCOF-12. **d** Zn-TDCOF-15.

Table S4. Scherrer analysis table of TDCOF and Zn-TDCOF.

Sample	2θ (degrees)	β (FWHM, radians)	τ (nm)
TDCOF	4.24	0.0068	22.7
Zn-TDCOF-6	4.25	0.0077	20.2
Zn-TDCOF-9	4.26	0.0081	19.0
Zn-TDCOF-12	4.28	0.0084	18.4
Zn-TDCOF-15	4.29	0.0094	16.4

The domain sizes were estimated by using the Scherrer equation:

$$\tau = \frac{k\lambda}{\beta\cos(\theta)}$$

where:

τ is the crystalline domain size (unit: nm);

k is shape factor with the value of 1.0;

λ = 0.154056 nm (Cu Kα X-ray wavelength);

β is the line broadening at half the maximum intensity (FWHM) of the (200) peak from PXRD in radians;

θ is the location of the observed peak from PXRD in degrees.

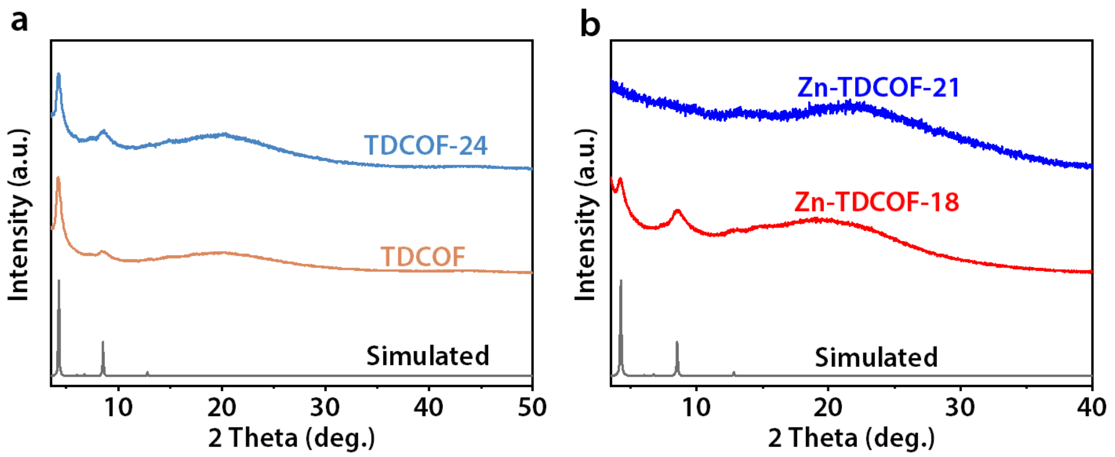

Fig. S21 PXRD patterns of TDCOF based on different treatment at 40 ^{o}C. **a** TDCOF with treatment for 24 h on the bare EtOH solution (TDCOF-24). **b** TDCOF with different treatment time (18 and 21 h) on EtOH solution of zinc acetate (Zn-TDCOF-18 and Zn-TDCOF-21).

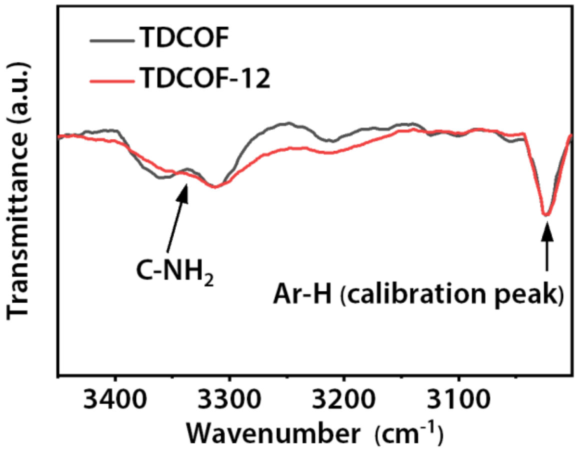

Fig. S22 FT-IR spectra of TDCOF was treated with similar method as Zn-TDCOF except that zinc salt is not added (TDCOF-12, bare EtOH solution).

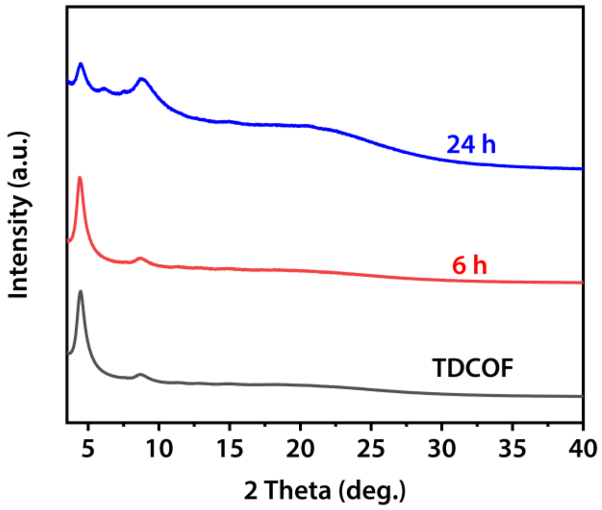

Fig. S23 PXRD patterns of TDCOF with different treatment time (6 and 24 h) on THF solution of zinc acetate.

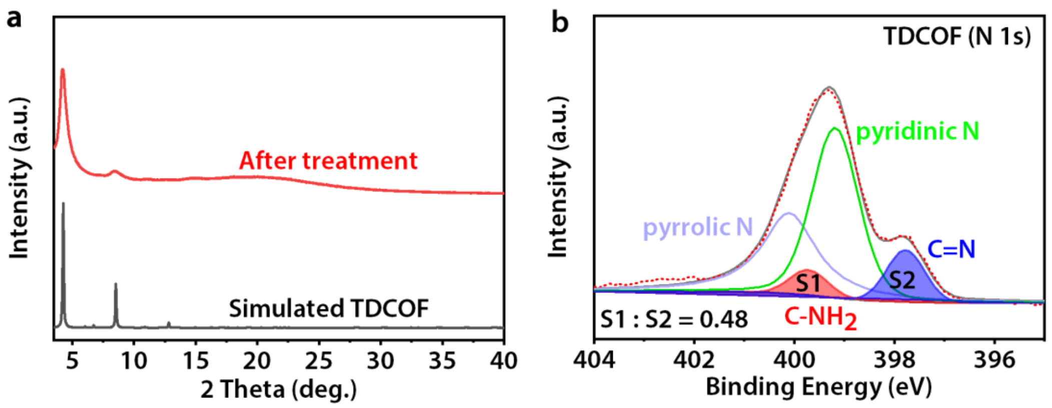

Fig. S24 Characterization of TDCOF after 12 h treatment in THF solution of anhydrous zinc acetate with similar method to Zn-TDCOF-12. **a** PXRD pattern. **b** XPS spectrum.

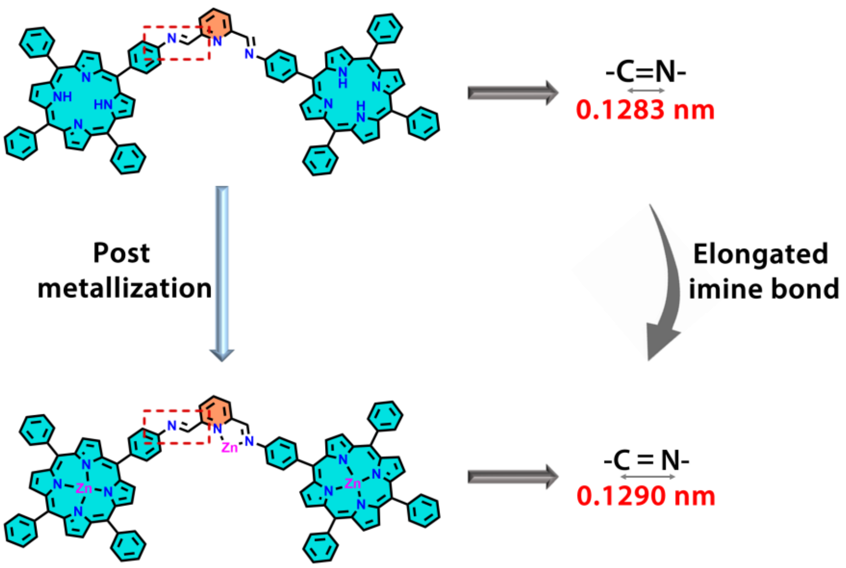

Fig. S25 Change of imine bond length after post-metallization is based on the DFT calculation.

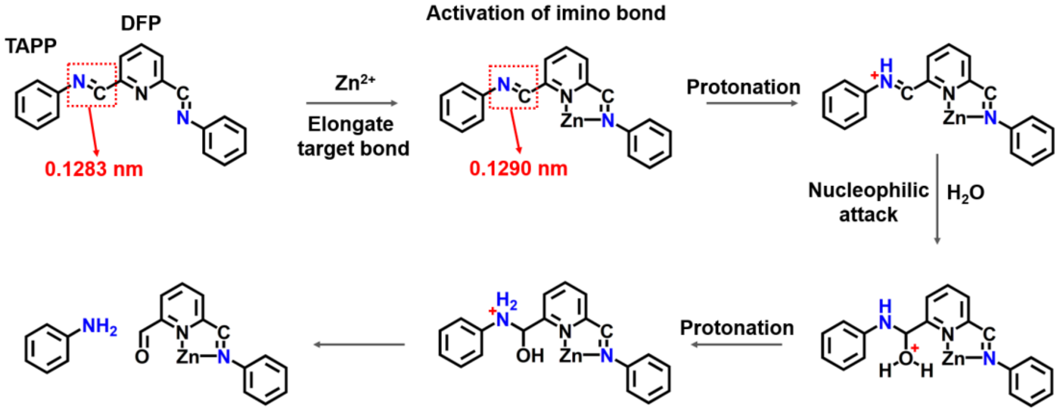

Fig. S26 Mechanism of Zn^{2+}-promoted imine linkage hydrolysis for TDCOF.

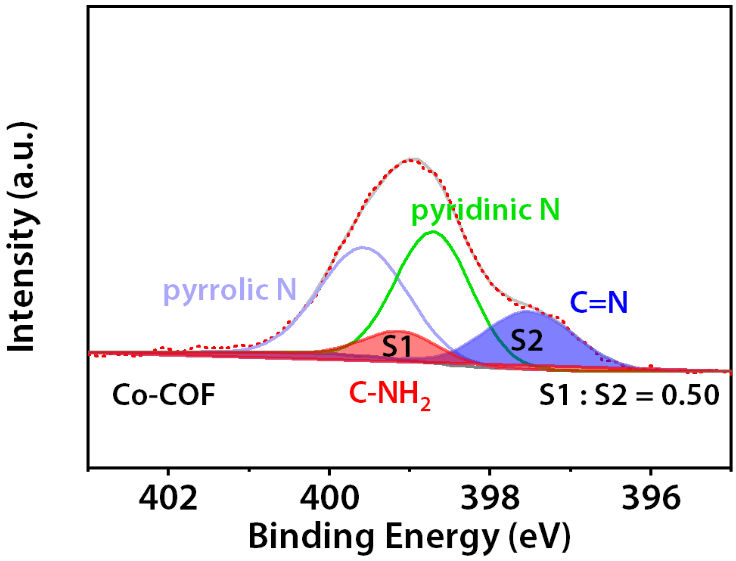

Fig. S27 N 1s XPS spectrum of Co-COF with semi-quantitative analysis for C-NH_2 and C=N.

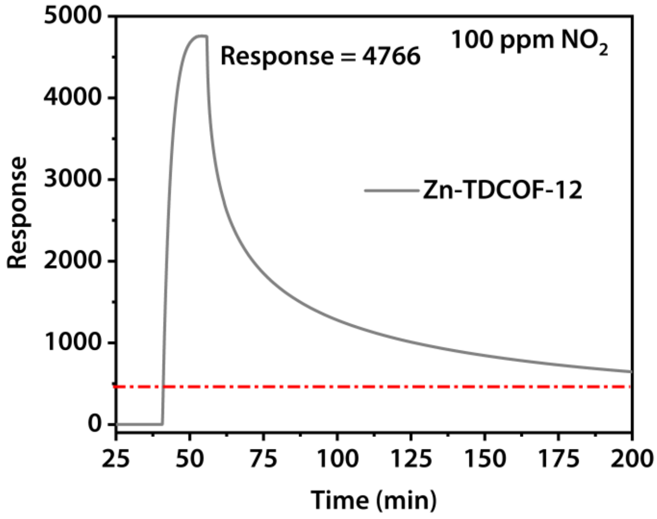

Fig. S28 Response-recovery curve toward 100 ppm NO_2 of Zn-TDCOF-12 under dark conditions.

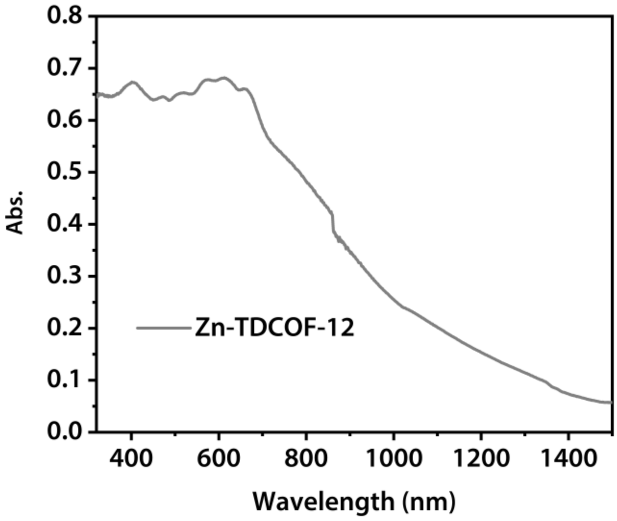

Fig. S29 Solid-state UV/Vis absorption spectra of Zn-TDCOF-12.

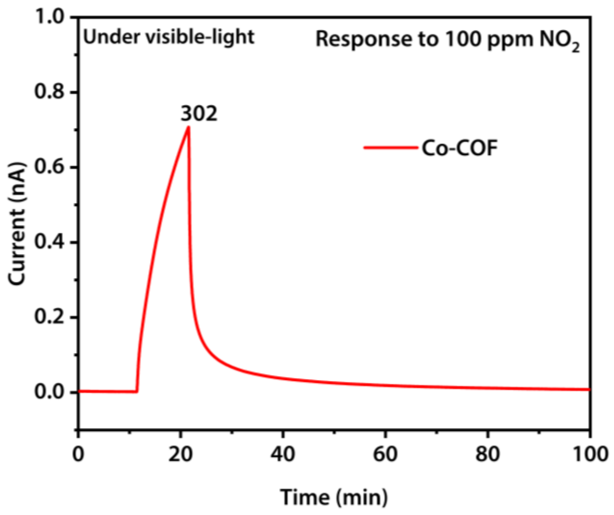

Fig. S30 Response-recovery curve toward 100 ppm NO_2 of Co-COF under dark visible light and room temperature.

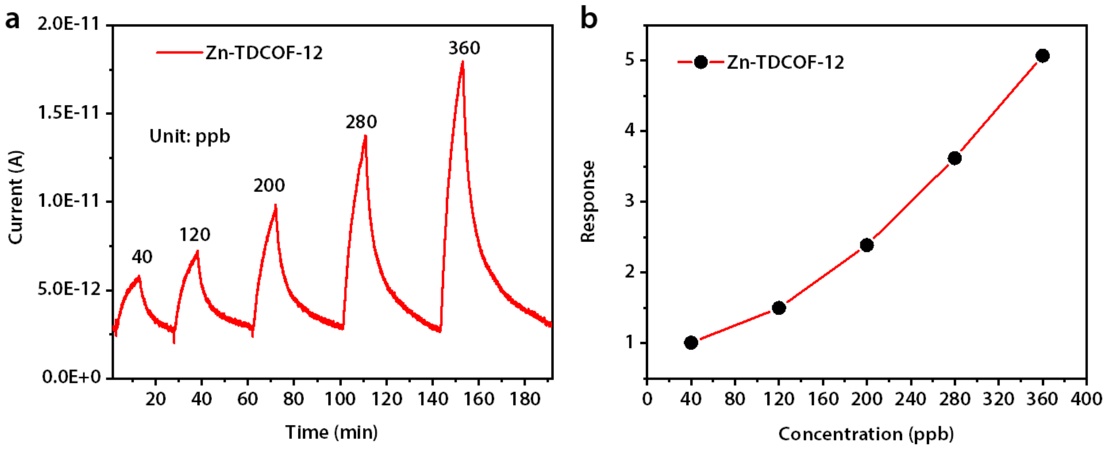

Fig. S31 NO_2 sensing of Zn-TDCOF-12 with the different concentrations of 40 - 360 ppb. **a** Response-recovery curve toward NO_2. **b** Sensitivity.

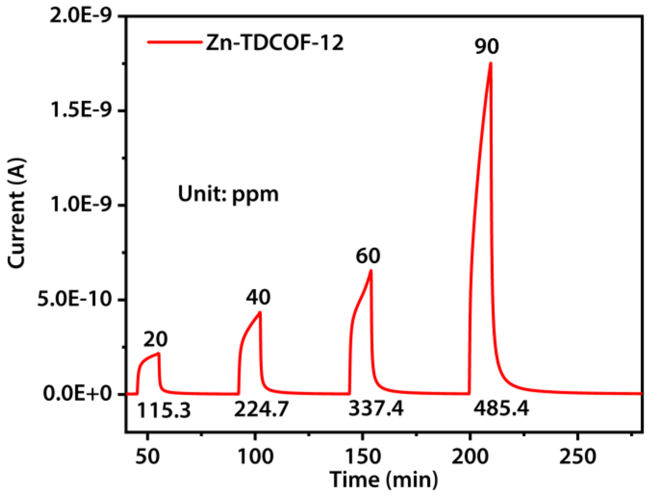

Fig. S32 Response-recovery curve toward NO_2 with the different concentrations (20 – 90 ppm) of Zn-TDCOF-12.

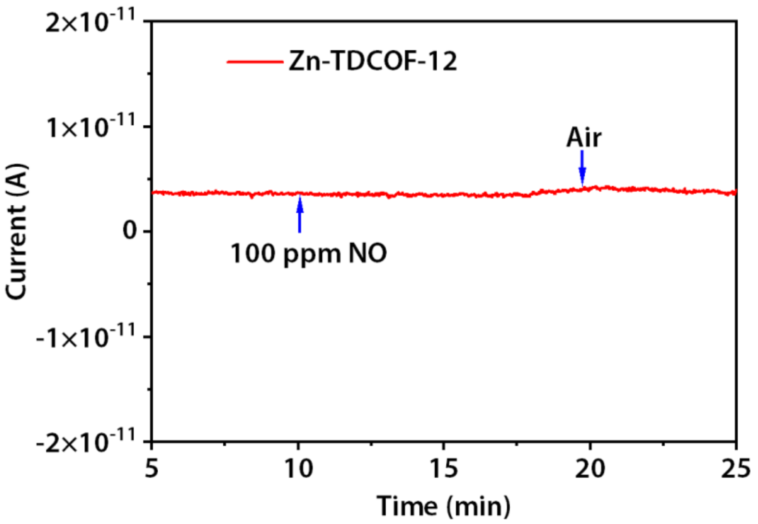

Fig. S33. Sensing current response to 100 ppm NO of Zn-TDCOF-12.

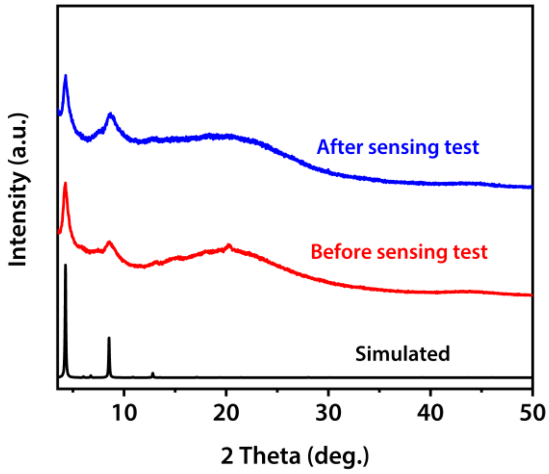

Fig. S34 Stability test of Zn-TDCOF-12 before and after the gas sensing test.

Table S5 Compared with reported materials for the NO_2 sensing under visible-light and room temperature.

	Concentration	Response	$t_{res.}/t_{rec.}$	Ref.
Au–MoS_2	50 ppm	32.5	-/27 s	10
Cu/Cu_2O	10 ppm	5.27	30 s/-	11
ZnO-Ag	1 ppm	1.1	3.3 min/4.2 min	12
Dye activated a-ZnO	10 ppm	5.9	2 h/14 h	13
H7	300 ppb	1.4	62 min/70 min	14
3D TiO_2	5 ppm	3.7	7.1 min/-	15
2D SnS_2 nanoflowers	5 ppm	14.3	12.2 min/62 min	16
S1	372 ppb	0.89	20.8 min/> 60 min	17
WO_3@GO	0.9 ppm	62.73	28.7 min/> 60 min	18
CdS–ZnO	1 ppm	30.9	> 50 min/-	19
$SnO_{1\text{-}@}ZnO_{1\text{-}\beta}$@$SnO_{2\text{-}\gamma}$	1 ppm	2.36	20.3 min/28.8 min	20
SnS_2 nanosheets	8 ppm	10.8	3.3 min/13.1 min	21
SnS_2/rGO	1 ppm	0.65	1.3 min/4.0 min	22
Fe_2O_3–$Cu_3(HHTP)_2$-NFs	5 ppm	0.89	-	23
reacted MoS_2	5 ppm	0.5	-	24
CdS nanoflake array	20 ppm	0.57	0.22 min/2.1 min	25
Au-SnO_2 (red light)	5 ppm	175	38.3 min/no recovery	26
SnO_2@SnS_2	0.2 ppm	5.2	15.8 min/19.3 min	27
MoS_2/GaSe	500 ppb	0.62	0.38 min/2.97 min	28
Co–ZnO	50 ppm	1.3	-	29
SnS_2/TiO_2	1 ppm	0.4	0.72 min/1.7 min	30
IGZO thin-film	5 ppm	12.5	-/10 min	31
2D/2D ZnO/g-C_3N_4 (460 nm)	7 ppm	44.8	2.8 min/6.1 min	32
ZnO nanorod/Au hybrids	10 ppm	17.5	25 s/29 s	33

ZnO/Pd hybrids	100 ppb	0.16	-/3.1 min	34
3D In_2O_3-ZnO	5 ppm	9	-	35
In_2O_3 nanowire array	500 ppb	4.5	9 min/20 min	36
MXene/WS_2	10 ppm	0.6	56 s/53 s	37
SV-MoS_2	200 ppb	0.45	2.9 min/5.4 min	38
3D CNC	50 ppm	9	1.5 min/1.7 min	39
CdS/ZnO	100 ppb	0.35	0.7 min/5.7 min	40
HOF-1	100 ppm	1700	2.5 min/0.6 min	41
**TDCOF**	**100 ppm**	**228**	**6.0 min/1.8 min**	**This work**
**Zn-TDCOF-12**	**100 ppm**	**554**	**8.1 min/10.6 min**	**This work**
**Zn-TDCOF-12**	**1 ppm**	**7.1**	**-**	**This work**
**Zn-TDCOF-15**	**100 ppm**	**252**	**8.6 min/8.9 min**	**This work**

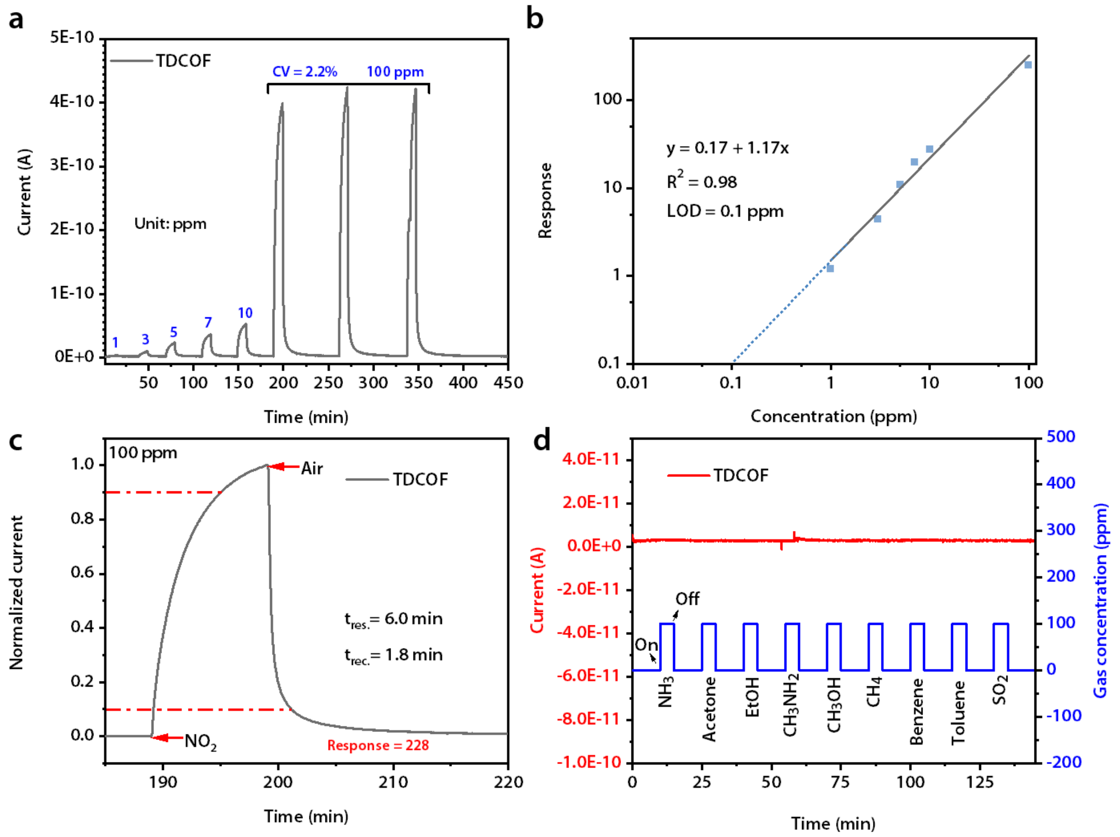

Fig. S35 Photo-induced gas sensor of TDCOF. **a** Response-recovery curve toward NO_2 with different concentrations. **b** Response-concentration log-log plots for the NO_2 sensor with different concentrations. **c** Response-recovery time curves to 100 ppm NO_2 with the response and recovery time were calculated. **d** Sensing current response to 9 types of interfering gases (100 ppm).

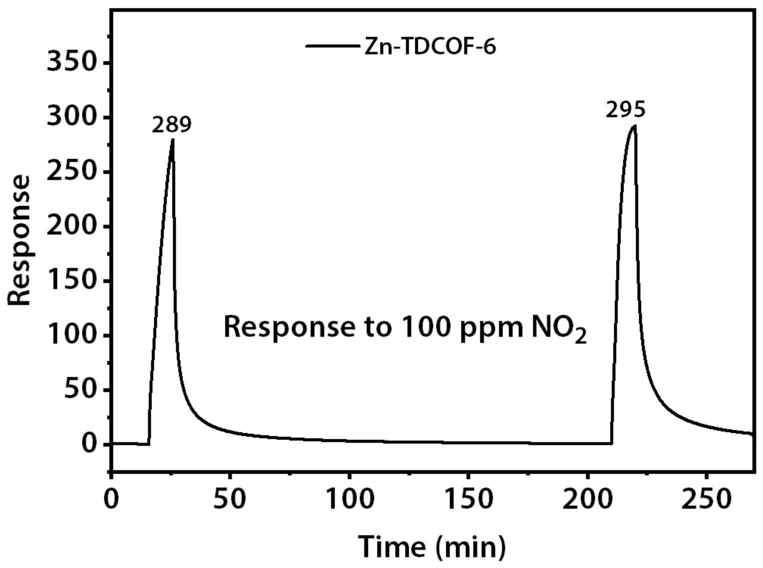

Fig. S36 Response-recovery curve toward 100 ppm NO_2 of Zn-TDCOF-6.

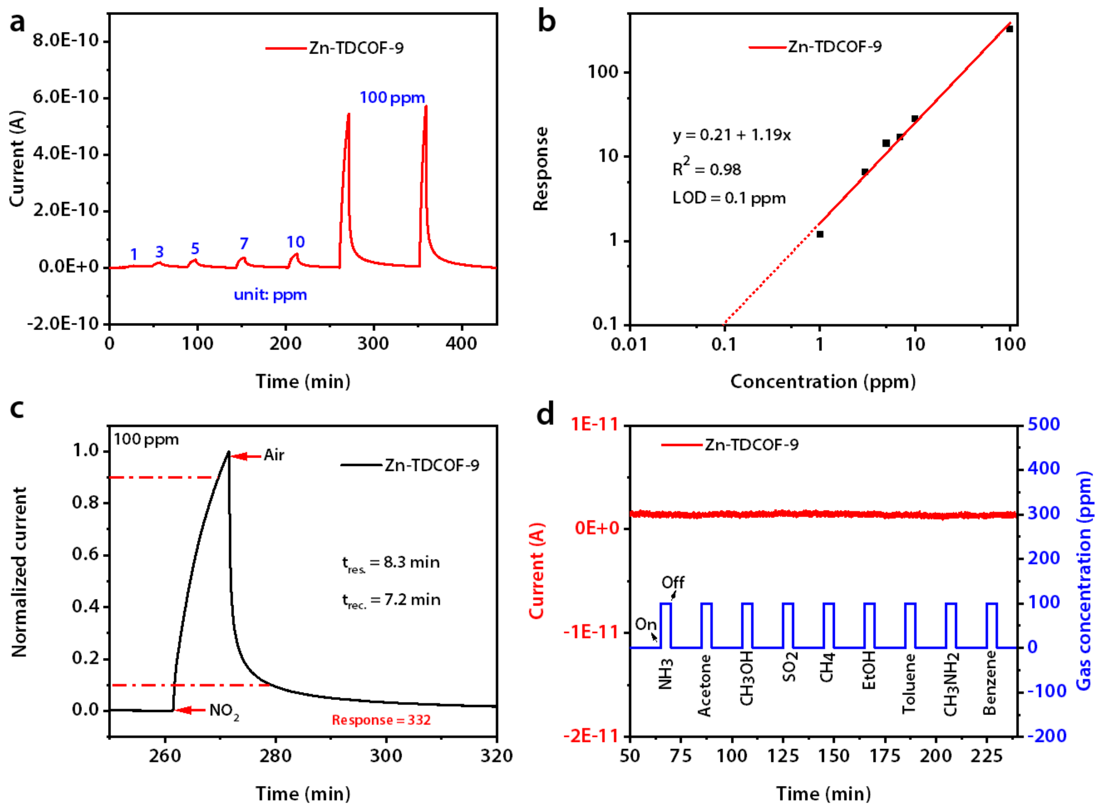

Fig. S37 Photo-induced gas sensor of Zn-TDCOF-9. **a** Response-recovery curve toward NO_2 with different concentrations. **b** Response-concentration log-log plots for the NO_2 sensor with different concentrations. **c** Response-recovery time curves to 100 ppm NO_2 with the response and recovery time were calculated. **d** Sensing current response to 9 types of interfering gases (100 ppm).

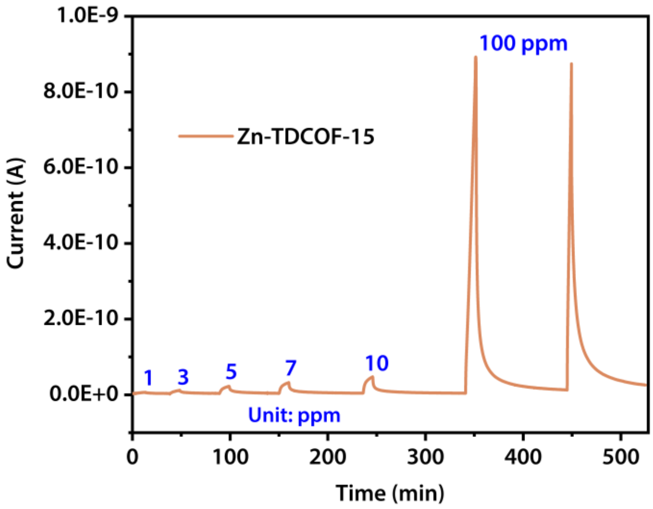

Fig. S38 Response-recovery curve toward NO_2 with different concentrations of Zn-TDCOF-15.

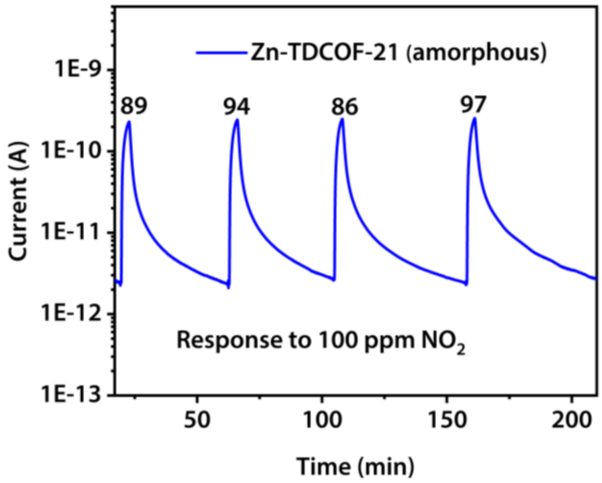

Fig. S39 Response-recovery curve toward 100 ppm NO_2 of Zn-TDCOF-21 (amorphous).

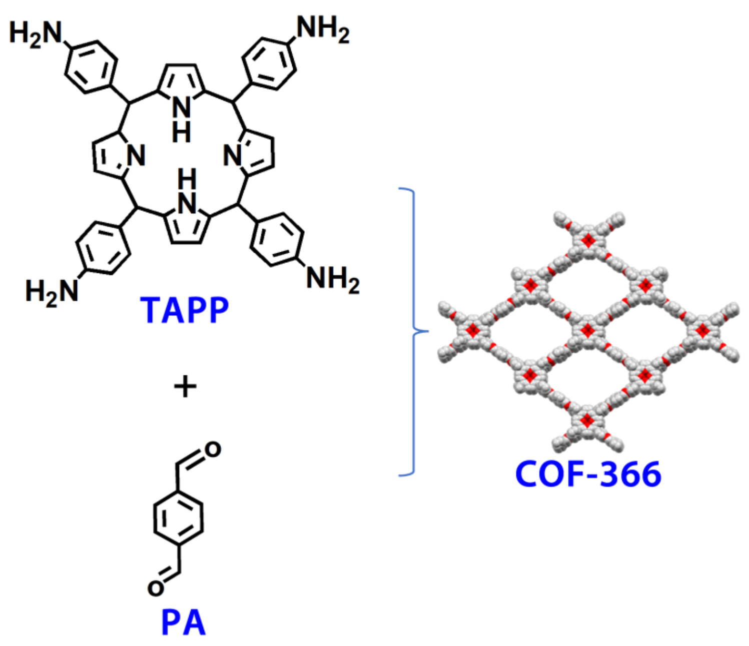

Fig. S40 Structure of COF-366.

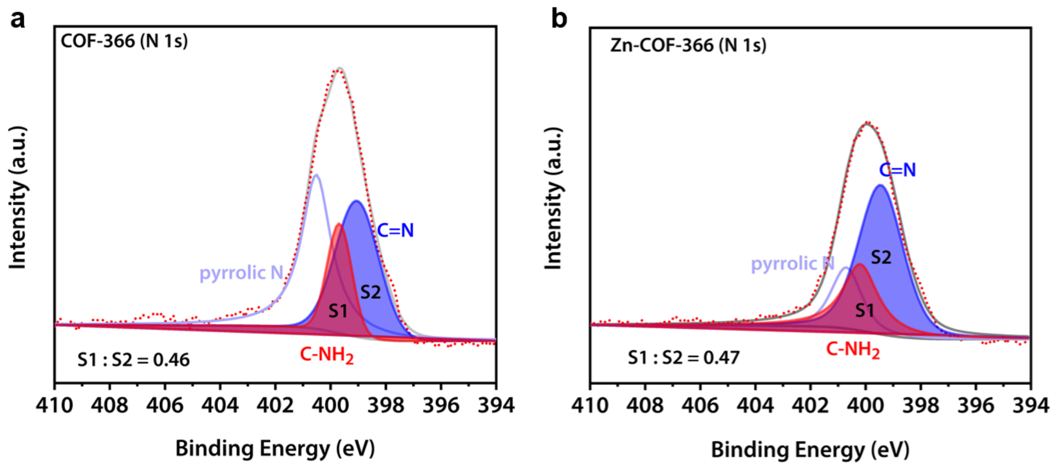

Fig. S41 N 1s XPS spectra of COF-366 and Zn-COF-366 with a ratio of C-NH_2 and C=N (dangling bond). **a** COF-366. **b** Zn-COF-366.

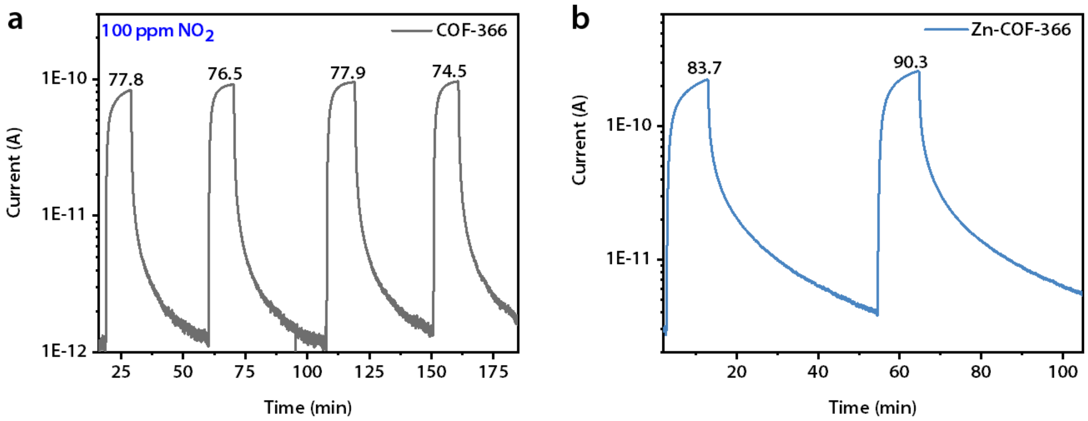

Fig. S42 Response-recovery curve toward 100 ppm NO_2 of COF-366 and Zn-COF-366 under visible light.

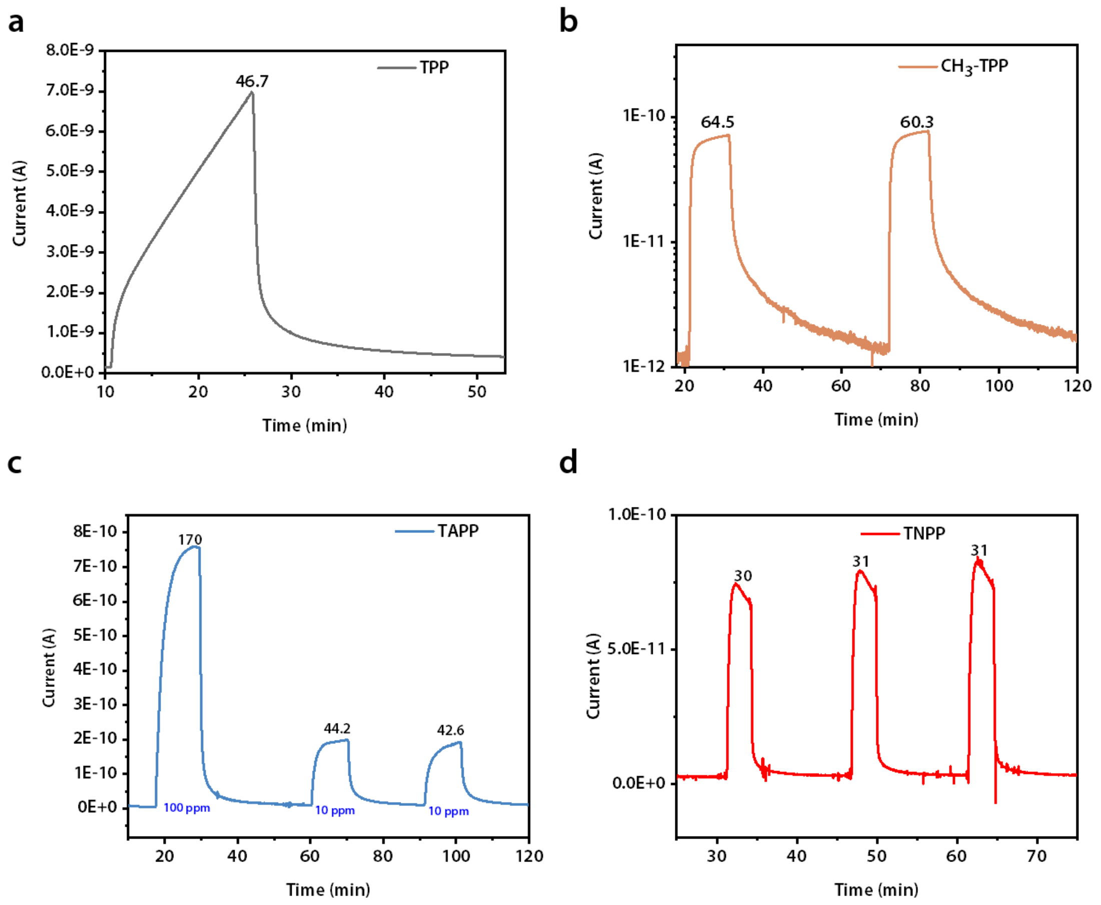

Fig. S43 Response-recovery curve toward 100 ppm NO_2 based on the contrast sample of TAPP with different functional groups under visible light. **a** TPP. **b** CH_3-TPP. **c** TAPP. **d** TNPP.

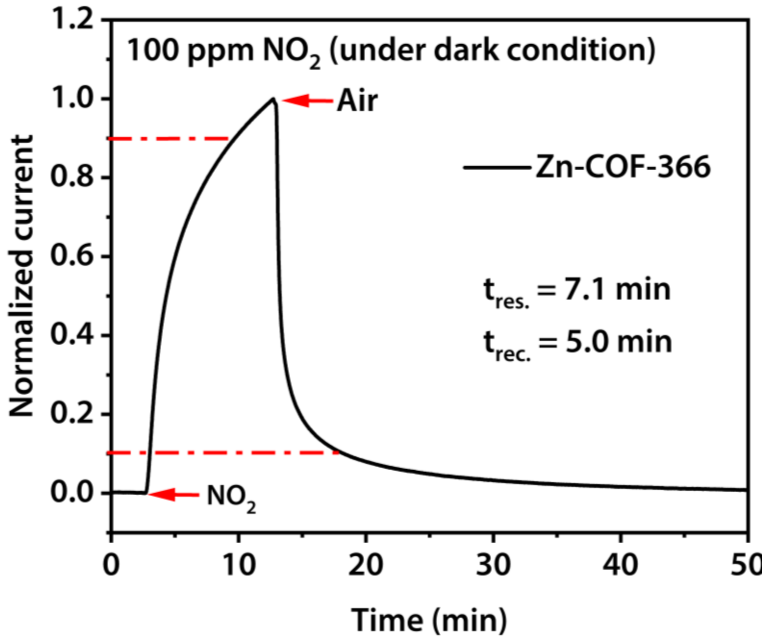

Fig. S44 Response-recovery time curves to 100 ppm NO_2 of Zn-COF-366 under dark conditions (π-backbonding to NO_2).

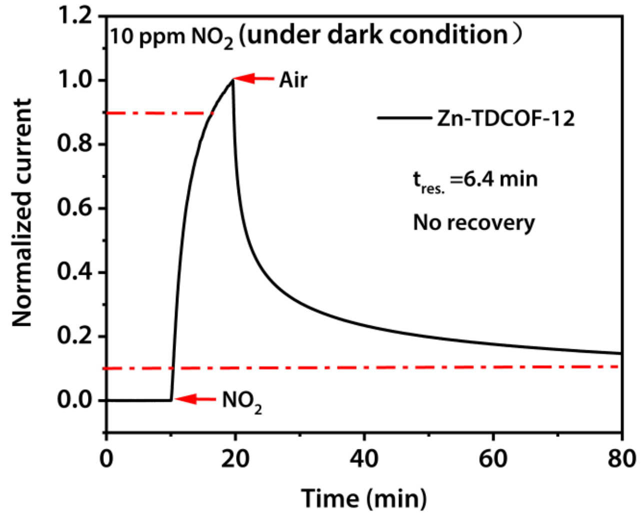

Fig. S45 Response-recovery time curves to 10 ppm NO_2 of Zn-TDCOF-12 under dark conditions (chemical adsorption to NO_2).

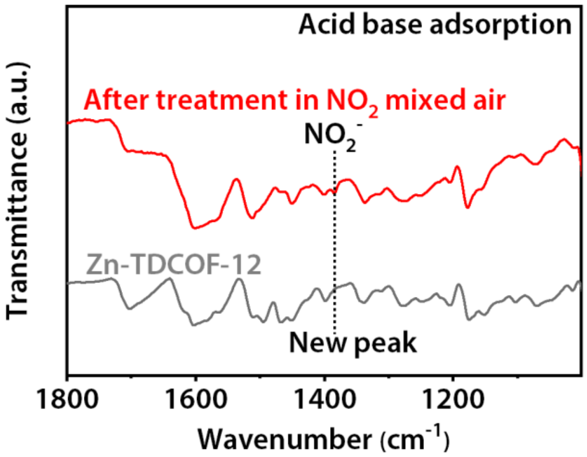

Fig. S46 FT-IR spectrum of Zn-TDCOF-12 after NO_2 (100 ppm NO_2, 2 h) treatment.

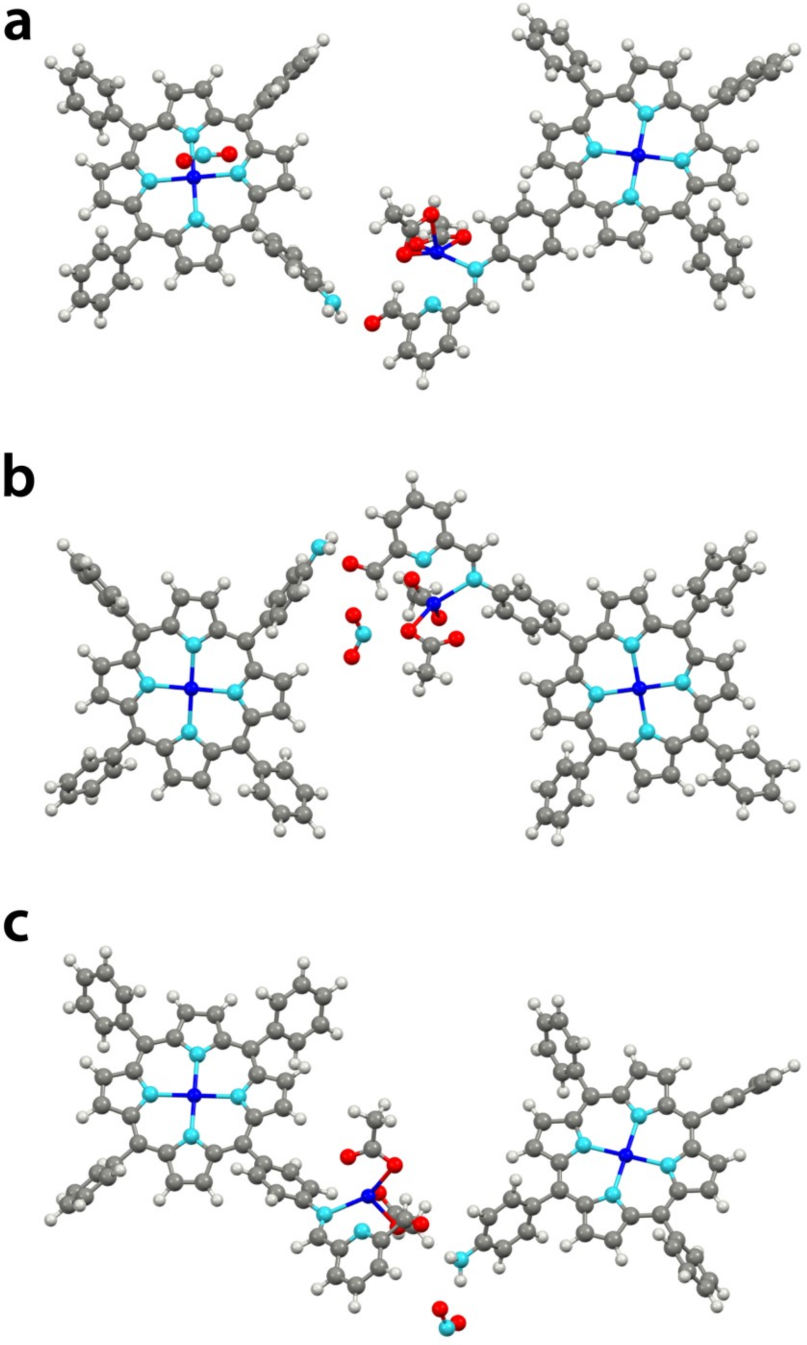

Fig. S47 Most favorable NO_2 adsorption configurations for the various sites of Zn-TDCOF. **a** Porphyrin zinc. **b** Pyridine zinc. **c** Dangling bond (-NH_2). The red, sky-blue, gray, white and navy-blue spheres represent O, N, C, H and Zn atoms, respectively.

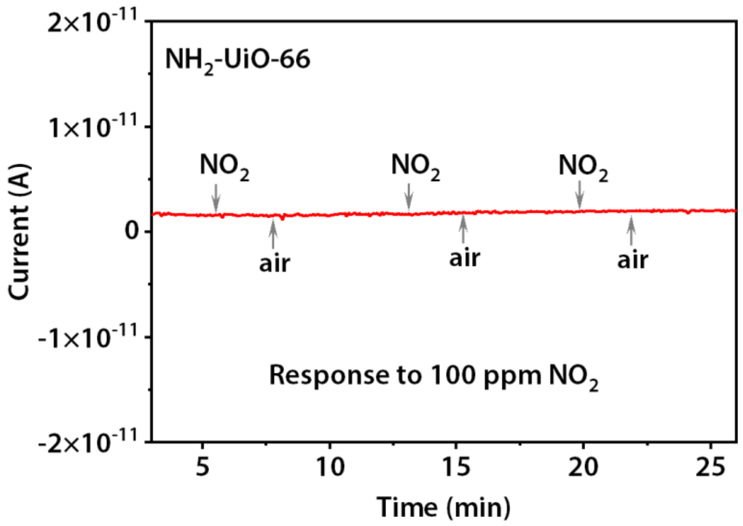

Fig. S48 Sensing current response to 100 ppm NO_2 of NH_2-UiO-66.

References

(1) Yao, M. S.; Tang, W. X.; Wang, G. E.; Nath, B.; Xu, G. MOF Thin Film-Coated Metal Oxide Nanowire Array: Significantly Improved Chemiresistor Sensor Performance. *Adv. Mater.* **2016**, *28* (26), 5229-5234.

(2) Delley, B. An all-electron numerical method for solving the local density functional for polyatomic molecules. *J. Chem. Phys.* **1990**, *92* (1), 508-517.

(3) Delley, B. From molecules to solids with the $DMol^3$ approach. *J. Chem. Phys.* **2000**, *113* (18), 7756-7764.

(4) Versluis, L.; Ziegler, T. The determination of molecular structures by density functional theory. The evaluation of analytical energy gradients by numerical integration. *J. Chem. Phys.* **1988**, *88* (1), 322-328.

(5) Barth, U. v.; Hedin, L. A local exchange-correlation potential for the spin polarized case. i. *J. Phys. C: Solid State Phys.* **1972**, *5* (13), 1629-1642.

(6) Lee, C.-S.; Hwang, T.-S.; Wang, Y.; Peng, S.-M.; Hwang, C.-S. Charge Density and Bonding in Bis(diiminosuccinonitrilo)nickel, $Ni(C_4N_4H_2)_2$: A Combined Experimental and Theoretical Study. *J. Phys. Chem.* **1996**, *100* (8), 2934-2941.

(7) Lin, T.; Zhang, W.-D.; Huang, J.; He, C. A DFT Study of the Amination of Fullerenes and Carbon Nanotubes: Reactivity and Curvature. *J. Phys. Chem. B* **2005**, *109* (28), 13755-13760.

(8) Matsuzawa, N.; Seto, J. e.; Dixon, D. A. Density Functional Theory Predictions of Second-Order Hyperpolarizabilities of Metallocenes. *J. Phys. Chem. A* **1997**, *101* (49), 9391-9398.

(9) Shang, S.; Yang, C.; Wang, C.; Qin, J.; Li, Y.; Gu, Q.; Shang, J. Transition-Metal-Containing Porphyrin Metal–Organic Frameworks as π-Backbonding Adsorbents for NO_2 Removal. *Angew. Chem. Int. Ed.* **2020**, *59* (44), 19680-19683.

(10) Chen, P.; Hu, J.; Yin, M.; Bai, W.; Chen, X.; Zhang, Y. MoS_2 Nanoflowers Decorated with Au Nanoparticles for Visible-Light-Enhanced Gas Sensing. *ACS Appl. Nano Mater.* **2021**, *4* (6), 5981-5991.

(11) Zou, X.; Fan, H.; Tian, Y.; Zhang, M.; Yan, X. Microwave-assisted hydrothermal synthesis of Cu/Cu_2O hollow spheres with enhanced photocatalytic and gas sensing activities at room temperature. *Dalton Trans.* **2015**, *44* (17), 7811-7821.

(12) Zhang, Q.; Xie, G.; Xu, M.; Su, Y.; Tai, H.; Du, H.; Jiang, Y. Visible light-assisted room temperature gas sensing with ZnO-Ag heterostructure nanoparticles. *Sens. Actuators, B* **2018**, *259*, 269-281.

(13) Zhang, C.; Wang, J.; Olivier, M.-G.; Debliquy, M. Room temperature nitrogen dioxide sensors based on N719-dye sensitized amorphous zinc oxide sensors performed under visible-light illumination. *Sens. Actuators, B* **2015**, *209*, 69-77.

(14) Zhang, C.; Liu, G.; Liu, K.; Wu, K. ZnO_{1-x} coatings deposited by atmospheric plasma spraying for room temperature ppb-level NO_2 detection. *Appl. Surf. Sci.* **2020**, *528*, 147041.

(15) Cho, D.; Suh, J. M.; Nam, S.-H.; Park, S. Y.; Park, M.; Lee, T. H.; Choi, K. S.; Lee, J.; Ahn, C.; Jang, H. W.; et al. Optically Activated 3D Thin-Shell TiO_2 for Super-Sensitive Chemoresistive Responses: Toward Visible Light Activation. *Adv. Sci.* **2021**, *8* (3), 2001883.

(16) Eom, T. H.; Cho, S. H.; Suh, J. M.; Kim, T.; Lee, T. H.; Jun, S. E.; Yang, J. W.; Lee, J.; Hong, S.-H.; Jang, H. W. Substantially improved room temperature NO_2 sensing in 2-dimensional SnS_2 nanoflowers enabled by visible light illumination. *J. Mater. Chem. A* **2021**, *9* (18), 11168-11178.

(17) Geng, X.; Lahem, D.; Zhang, C.; Li, C.-J.; Olivier, M.-G.; Debliquy, M. Visible light enhanced black NiO sensors for ppb-level NO_2 detection at room temperature. *Ceram. Int.* **2019**, *45* (4), 4253-4261.

(18) Geng, X.; You, J.; Wang, J.; Zhang, C. Visible light assisted nitrogen dioxide sensing using tungsten oxide - Graphene oxide nanocomposite sensors. *Mater. Chem. Phys.* **2017**, *191*, 114-120.

(19) Geng, X.; Zhang, C.; Debliquy, M. Cadmium sulfide activated zinc oxide coatings deposited by liquid plasma spray for room temperature nitrogen dioxide detection under visible light illumination. *Ceram. Int.* **2016**, *42* (4),

4845-4852.
(20) Geng, X.; Zhang, C.; Luo, Y.; Liao, H.; Debliquy, M. Light assisted room-temperature NO_2 sensors with enhanced performance based on black $SnO_{1-\alpha}$@$ZnO_{1-\beta}$@$SnO_{2-\gamma}$ nanocomposite coatings deposited by solution precursor plasma spray. *Ceram. Int.* **2017**, *43* (8), 5990-5998.
(21) Gu, D.; Wang, X.; Liu, W.; Li, X.; Lin, S.; Wang, J.; Rumyantseva, M. N.; Gaskov, A. M.; Akbar, S. A. Visible-light activated room temperature NO_2 sensing of SnS_2 nanosheets based chemiresistive sensors. *Sens. Actuators* **2020**, *305*, 127455.
(22) Huang, Y.; Jiao, W.; Chu, Z.; Ding, G.; Yan, M.; Zhong, X.; Wang, R. Ultrasensitive room temperature ppb-level NO_2 gas sensors based on SnS_2/rGO nanohybrids with P-N transition and optoelectronic visible light enhancement performance. *J. Mater. Chem. C* **2019**, *7* (28), 8616-8625.
(23) Jo, Y.-M.; Lim, K.; Yoon, J. W.; Jo, Y. K.; Moon, Y. K.; Jang, H. W.; Lee, J.-H. Visible-Light-Activated Type II Heterojunction in Cu_3(hexahydroxytriphenylene)$_2$/Fe_2O_3 Hybrids for Reversible NO_2 Sensing: Critical Role of π–π* Transition. *ACS Cent. Sci.* **2021**, *7* (7), 1176-1182.
(24) Kang, Y.; Pyo, S.; Jo, E.; Kim, J. Light-assisted recovery of reacted MoS_2 for reversible NO_2 sensing at room temperature. *Nanotechnology* **2019**, *30* (35), 355504.
(25) Li, H.-Y.; Yoon, J.-W.; Lee, C.-S.; Lim, K.; Yoon, J.-W.; Lee, J.-H. Visible light assisted NO_2 sensing at room temperature by CdS nanoflake array. *Sens. Actuators, B* **2018**, *255*, 2963-2970.
(26) Lim, K.; Jo, Y.-M.; Yoon, J.-W.; Kim, J.-S.; Lee, D.-J.; Moon, Y. K.; Yoon, J. W.; Kim, J.-H.; Choi, H. J.; Lee, J.-H. A Transparent Nanopatterned Chemiresistor: Visible-Light Plasmonic Sensor for Trace-Level NO_2 Detection at Room Temperature. *Small* **2021**, *17* (20), 2100438.
(27) Liu, D.; Tang, Z.; Zhang, Z. Visible light assisted room-temperature NO_2 gas sensor based on hollow SnO_2@SnS_2 nanostructures. *Sens. Actuators, B* **2020**, *324*, 128754.
(28) Niu, Y.; Zeng, J.; Liu, X.; Li, J.; Wang, Q.; Li, H.; de Rooij, N. F.; Wang, Y.; Zhou, G. A Photovoltaic Self-Powered Gas Sensor Based on All-Dry Transferred MoS_2/GaSe Heterojunction for ppb-Level NO_2 Sensing at Room Temperature. *Adv. Sci.* **2021**, *8* (14), 2100472.
(29) Sreedhar, A.; Reddy, I. N.; Ta, Q. T. H.; Doan, T. H. P.; Shim, J.; Noh, J.-S. Unveiling the impact of interfacially engineered selective V_2O_5 nanobelt bundles with flake-like ZnO and Co-ZnO thin films for multifunctional visible-light water splitting and toxic gas sensing. *J. Power Sources* **2020**, *478*, 229081.
(30) Sun, Q.; Li, Y.; Hao, J.; Zheng, S.; Zhang, T.; Wang, T.; Wu, R.; Fang, H.; Wang, Y. Increased Active Sites and Charge Transfer in the SnS_2/TiO_2 Heterostructure for Visible-Light-Assisted NO_2 Sensing. *ACS Appl. Mater. Interfaces* **2021**, *13* (45), 54152-54161.
(31) Vijjapu, M. T.; Surya, S. G.; Yuvaraja, S.; Zhang, X.; Alshareef, H. N.; Salama, K. N. Fully Integrated Indium Gallium Zinc Oxide NO_2 Gas Detector. *ACS Sens.* **2020**, *5* (4), 984-993.
(32) Wang, H.; Bai, J.; Dai, M.; Liu, K.; Liu, Y.; Zhou, L.; Liu, F.; Liu, F.; Gao, Y.; Yan, X.; et al. Visible light activated excellent NO_2 sensing based on 2D/2D ZnO/g-C_3N_4 heterojunction composites. *Sens. Actuators, B* **2020**, *304*, 127287.
(33) Wang, J.; Fan, S.; Xia, Y.; Yang, C.; Komarneni, S. Room-temperature gas sensors based on ZnO nanorod/Au hybrids: Visible-light-modulated dual selectivity to NO_2 and NH_3. *J. Hazard. Mater.* **2020**, *381*, 120919.
(34) Wang, J.; Hu, C.; Xia, Y.; Komarneni, S. Highly sensitive, fast and reversible NO_2 sensors at room-temperature utilizing nonplasmonic electrons of ZnO/Pd hybrids. *Ceram. Int.* **2020**, *46* (6), 8462-8468.
(35) Wang, T.; Yu, Q.; Zhang, S.; Kou, X.; Sun, P.; Lu, G. Rational design of 3D inverse opal heterogeneous composite microspheres as excellent visible-light-induced NO_2 sensors at room temperature. *Nanoscale* **2018**, *10* (10), 4841-4851.
(36) Wang, X.-X.; Li, H.-Y.; Guo, X. Flexible and transparent sensors for ultra-low NO_2 detection at room

temperature under visible light illumination. *J. Mater. Chem. A* **2020**, *8* (29), 14482-14490.

(37) Xia, Y.; He, S.; Wang, J.; Zhou, L.; Wang, J.; Komarneni, S. MXene/WS_2 hybrids for visible-light-activated NO_2 sensing at room temperature. *Chem. Commun.* **2021**, *57* (72), 9136-9139.

(38) Xia, Y.; Hu, C.; Guo, S.; Zhang, L.; Wang, M.; Peng, J.; Xu, L.; Wang, J. Sulfur-Vacancy-Enriched MoS_2 Nanosheets Based Heterostructures for Near-Infrared Optoelectronic NO_2 Sensing. *ACS Appl. Nano Mater.* **2020**, *3* (1), 665-673.

(39) Yang, F.; Zheng, Z.; Lin, Z.; Wang, B.; Liu, P.; Yang, G. Visible-light-driven room-temperature gas sensor based on carbyne nanocrystals. *Sens. Actuators, B* **2020**, *316*, 128200.

(40) Yang, Z.; Guo, L.; Zu, B.; Guo, Y.; Xu, T.; Dou, X. CdS/ZnO Core/Shell Nanowire-Built Films for Enhanced Photodetecting and Optoelectronic Gas-Sensing Applications. *Adv. Optical Mater.* **2014**, *2* (8), 738-745.

(41) Deng, W.-H.; He, L.; Chen, E.-X.; Wang, G.-E.; Ye, X.-L.; Fu, Z.-H.; Lin, Q.; Xu, G. Crystalline microporous small molecule semiconductors based on porphyrin for high-performance chemiresistive gas sensing. *J. Mater. Chem. A* **2022**, *10* (24), 12977-12983.
